# Polymorphic variations and mRNA expression of the genes encoding interleukins as well as enzymes of oxidative and nitrative stresses as a potential risk of nephrolithiasis development

**DOI:** 10.1371/journal.pone.0293280

**Published:** 2023-10-25

**Authors:** Paulina Wigner-Jeziorska, Radosław Grębowski, Joanna Saluk, Michał Bijak, Janusz Szemraj

**Affiliations:** 1 Department of General Biochemistry, Faculty of Biology and Environmental Protection, University of Lodz, Lodz, Poland; 2 Department of Medical Biochemistry, Medical University of Lodz, Lodz, Poland; 3 Department of Urology, Provincial Integrated Hospital in Płock, Plock, Poland; 4 Biohazard Prevention Centre, Faculty of Biology and Environmental Protection, University of Lodz, Lodz, Poland; China Medical University, TAIWAN

## Abstract

Urolithiasis is one of the most common urological diseases worldwide with an unclear aetiology. However, a growing body of evidence suggests the potential role of molecular disturbances of the inflammation as well as oxidative and nitrative stresses, in the pathogenesis of urolithiasis. Therefore, we aimed to detect the potential association between six selected single-nucleotide polymorphisms (SNPs) and the development of nephrolithiasis. Moreover, we verified the association of urolithiasis development and mRNA expression of *IL-6*, *IL-8*, *SOD2*, and *NOS2* in peripheral blood mononuclear cells (PBMCs). Total genomic DNA and mRNA were isolated from the peripheral blood of 112 patients with urolithiasis and 114 healthy subjects. Using Taq-Man® probes, we genotyped the following SNPs: rs1800797 and rs2069845 in *IL-6*, rs2227307 in *IL-8*, rs4880 in *SOD2*, rs2297518 and rs2779249 in *NOS2*. In turn, the evaluation of mRNA expression was performed using real-time PCR and 2^-ΔCt^ methods. We found that the C/T genotype of the c.47 T>C–*SOD2* SNP increased the frequency of urolithiasis occurrence whereas the T/T homozygote of the same polymorphism decreased the risk of urolithiasis development in the Polish population. Moreover, our study confirmed that patients with urolithiasis were characterised by decreased *IL-6*, *IL-8*, and *SOD2* mRNA expression levels compared to the controls. In conclusion, our results suggest that polymorphic variants and changes in mRNA expression of *IL-6*, *IL8*, *SOD2*, and *NOS2* may be involved in the pathophysiology of urolithiasis.

## Introduction

The change in the lifestyle of modern society (poor diet and reduced physical activity) has consequently led to a significant increase in the incidence of urolithiasis in the last three decades. This disease consists of the deposition in the urinary tract of unsolved deposits of substances present in the urine [1–3]. Nowadays, nephrolithiasis takes third place on the list of the most common urological diseases, right after urinary tract infections and prostate diseases [2, 3]. Estimations show that the prevalence rates of urolithiasis are 1.7–14.8% and still rising [4]. Interestingly, nephrolithiasis is much more common in men than in women, however, its prevalence ratio has levelled out during the last decade [5]. This increase is due to the change in women’s lifestyle towards that of men, i.e. an increase in obesity and smoking [6]. Moreover, as many as 30–50% of people after the first attack of renal colic may experience a recurrence of the disease [7]. Subsequent episodes of urolithiasis contribute to the development of serious complications, including kidney failure and even cancer of the urinary system [8–10]. On the other hand, urinary stone formers are characterised by an increased risk of other systemic diseases, such as myocardial infarction, independent of chronic kidney disease [11].

Epidemiological analysis indicates that environmental factors are basic for the aetiology of urolithiasis, but the exact development mechanism of the disease still residues unrevealed. In addition to the male gender, increased BMI, obesity, high consumption of animal protein, sodium (salt), and sugar, diminished fluid high oxalate content in the diet and reduced physical activity should be mentioned among risk factors of the disease [12–17]. Nevertheless, as numerous studies have shown, the formation of the renal stone is a multifactorial process depending on environmental, anatomical, and genetic factors. Anatomical and genetic factors include the medullary sponge kidney, the horseshoe kidney, autosomal dominant polycystic kidney disease, and gout [18]. The genetic factors underlying plaque-forming hypercalciuria remain unexplained, but data from epidemiological studies suggest that approximately 20% of patients with idiopathic hypercalciuria have a family history of stones [19]. Interestingly, the study by Stechman et al. (2007) on twins also confirmed the strong heritability of hypercalciuria [20]. Moreover, a growing body of evidence highlights the potential role of molecular disturbances of the biochemical pathways, including oxidative stress and inflammation, in the pathogenesis of urolithiasis. In the course of urolithiasis, deposits of insoluble urine compound can adhere to the surface of renal tubular cells and then be internalised into cells by macropinocytosis for elimination. The end products of urinary stone degradation include, for example, Ca^2+^, which increases the intracellular calcium pool. Calcium overload of cells can lead to mitochondrial dysfunction and ROS overproduction. Moreover, crystal aggregation and retention in the urinary tract contribute to renin upregulation and angiotensin II overproduction, which consequently leads to NADPH oxidase activation, and thereby, additionally intensification of ROS generation. Previous research has shown that oxalate, the main component of urinary stones, can disrupt the electron transport chain in mitochondria, and therefore may lead to the leakage of free radicals [21]. It has been shown that as a result of exposure to oxalates, mitochondria increase the production of ROS, lipid peroxides and oxidised thiol proteins [22]. Calcium oxalate crystal deposition can also cause mitochondrial damage through increased cellular ceramide levels. The consequence of mitochondrial damage by ceramides may be increased production of hydrogen peroxide, glutathione depletion and decreased mitochondrial membrane potential, which results in the activation of caspases and ultimately the induction of apoptosis. The above-mentioned overproduction of hydrogen peroxide is the result of the increased activity of SOD2, neutralizing the superoxide anion radical generated in significant amounts as a result of the disturbed respiratory chain [23]. However, a prolonged state of oxidative stress may lead to the depletion of antioxidant defence enzymes, including SOD2. Moreover, in addition to increased oxidative stress, intensification of nitrosative stress processes was also observed in the course of urolithiasis. An animal study showed that kidney stones increase the level of nitrotyrosine, which may be a consequence of increased expression of inducible nitric oxide synthetase (iNOS) [24].

Interestingly, the already mentioned high ROS level activates transcription factors through the P38 mitogen-activated protein kinase (MAPK)/JNK signalling pathway, including nuclear factor kappa-light chain enhancer of activated B cells (NF-κB). In turn, ROS-induced NF- κB can regulate the expression of genes encoding pro-inflammatory cytokines, including tumour necrosis factor alpha (TNF-α), interleukin 6 (IL-6), interleukin 8 (IL-8), and C- reactive protein (CRP). In turn, the increased generation of pro-inflammatory cytokines, TNF-α, IL-6, IL-8 and CRP in a vicious circle mechanism may additionally activate NADPH oxidase and therefore may stimulate further ROS production [23, 25, 26]. As a consequence, the prolonged oxidative stress and inflammation observed especially in people with recurrent attacks of renal colic may induce damage to the urothelial cells and thus lead to severe complications, including the development of urinary tract neoplasms [8, 9].

Based on the above findings, oxidative and nitrosative stress as well as inflammation can be directly involved in urolithiasis pathogenesis and could be able to constitute a novel target for developing potential prognostic markers used in disease prevention. Thus, we aimed to find the association between the occurrence of polymorphisms located in genes encoding interleukin and antioxidant enzymes (-597 A>G (rs1800797) and c.3331 G>A (rs2069845) in *IL-6* gene, c.+396 T>G (rs2227307) in *IL-8*, (c.47 T>C (rs4880) in the *SOD2* gene, as well as nitric oxide synthetases c.1823C>T (rs2297518) and g.-1026 C>A (rs2779249) in the *NOS2* gene) and the development of urolithiasis as well as determining the impact of the urolithiasis development on the level of mRNA expression of appropriate genes, which will consequently contribute to expanding knowledge about the molecular pathogenesis of urolithiasis

## Materials and methods

### Participants

226 samples were collected from the 112 patients with urolithiasis (36 women and 76 men; mean age 56.31 ± 14.36) hospitalised at the Department of Urology of the Provincial Integrated Hospital in Plock, Poland and 114 sex-matched voluntary healthy donors without urolithiasis and who had no family history of the stone disease (control group; 39 women and 75 men; mean age 66.71 ± 11.76). The recruitment period for this study covered November 16, 2021, to December 20, 2022. The qualification procedure was the same as described in our publication [27]. Nevertheless, additional exclusion criteria were applied which included: using drugs such as estrogens, progesterone, glucocorticoids, diuretics, anticonvulsants, vitamin D, antiacid drugs, heparin, prostaglandin preparations, etc. In the case of the control group, immobilization for more than 2 months during the last 5 years, prolonged corticosteroid therapy (>3 months), alcohol consumption, vitamin D insufficiency and secondary hyperparathyroidism, metabolic acidosis, steroid, and anticonvulsant drug usage, other urinary system diseases, previous or current neoplastic diseases, autoimmune disorders. Additionally, all participants were asked to complete the same structural questionnaire from previously published articles to determine demographic and potential risk factors for urolithiasis, including age, lifestyle habits (e.g. g. the amount of fluids consumed daily, including coffee, type of diet), including smoking, body mass index (BMI), and co-occurrence disease (e. g. hypertension, diabetes, hypercholesterolemia) [27]. As in our previous studies [27, 28], the participation in the study had voluntary character and qualified individuals were native Poles from central Poland (not related) and were selected randomly without replacement sampling. Importantly, before deciding to participate in the experiment, all individuals were informed of the purpose and assured of the voluntary nature of the experiment and guaranteed that their personal data would be kept secret. Finally, all participants gave their written informed consent to participate in this study. The Bioethics Committee of the Faculty of Biology and Environmental Protection of the University of Lodz, Poland (approval no. 12/KBBN-UŁ/II/2020-21) and the Bioethics Committee of the Medical University of Lodz (no. RNN/141/21/KE) approved the research protocol. Moreover, the use of human samples was in line with the requirements of the Helsinki Declaration. The validity and reliability of the questionnaires were checked whenever possible. Detailed characteristics of patients and controls are presented in Results Section.

### Selection of single-nucleotide polymorphisms

The selection of SNPs localised in inflammation, as well as ROS and RNS (reactive nitrogen species) genes, was made according to the procedure described previously by our team [25, 26]. Finally, we chosen six SNPs, including -597 A>G (rs1800797) and c.3331 G>A (rs2069845) in *IL-6*, c.+396 T>G (rs2227307) in *IL-8*, c.47 T>C (p.Val16Ala) (rs4880) in *SOD2*, c.1823 C>T (p.Ser608Leu) (rs2297518) and g.-1026 C>A (rs2779249) in *NOS2*. Detailed information about studied polymorphism is presented in Table 1.

**Table 1:** Characteristics of all studied polymorphisms.

Gene	Polymorphism and NCBI db SNP ID (rs number)	Chromosome location	Region	Function	MAF in the European population	References
*IL-6*	-597 A>G (rs1800797)	7p15.3	Promoter	G allele carriers were characterised by increased inflammatory responses	G: 0.609	[29]
	c.3331 G>A (rs2069845)	7p15.3	Intron	G allele was associated with increased IL-6 secretion in serum	A: 0.539	[30]
*IL-8*	c.+396 T>G (rs2227307)	4q13.3	Intron	The modulation of IL-8 production	G: 0.447	[31]
*SOD2*	c.47 T>C (p.Val16Ala) (rs4880)	6q25.3	Exon	The substitution of Val to Ala led to functional modulation of protein and Val-carriers were characterised by reduced enzymatic activity	C: 0.498	[32]
*NOS2* (*iNOS*)	c.1823 C>T (p.Ser608Leu) (rs2297518)	17q11.2	Exon	The substitution from serine to leucine may contribute to increased iNOS activity	T: 0.198	[33]
	g.-1026 C>A (rs2779249)	17q11.2	Exon	A carriers were characterised by elevated *iNOS* promoter transcriptional activity	A: 0.295	[33]

### Blood sample collection, DNA and RNA isolation

After qualifying for participation in the study (in the years 2021–2022) and giving written consent to participate in the study, whole blood was collected from each participant from the cubital vein into a properly coded tube and stored at -20°C until the isolation of the genetic material. Genomic DNA and total RNA were isolated with a commercial kit—DNA/RNA Extracol Kit (EURX, Gdansk, Poland) as previously described by Grębowski et al. (2023) [27].

### SNPs genotyping

After the isolation of the genetic material from whole blood, we proceeded to genotype all the samples for all chosen genetic variants according to the protocol described in previous our publications [27, 28]. Briefly, real-time PCR was performed in a CFX96™ Real-Time PCR Detection System Thermal Cycler (Bio-Rad Laboratories, Inc., Hercules, CA, USA), using the TaqMan allele-specific discrimination assay, TaqMan® SNP Genotyping Assay (Thermo Fisher Scientific, Waltham, MA, USA), and RT PCR Mix Probe (A&A Biotechnology, Gdynia, Poland). Moreover, the CFX Manager TM Software (version 3.1) was used to analyse the obtained results. TaqMan® SNP Genotyping Assay details are included in Table 2.

**Table 2:** TaqMan® SNP genotyping Assay (Thermo Fisher Scientific, Waltham, MA, USA) was used in this study.

Polymorphism	Gene	Assay ID	Location
rs1800797	*IL-6*	C___1839695_20	Chr.7: 22726602
rs2069845		C___1839699_10	Chr.7: 22730530
rs2227307	*IL-8*	C__11748168_10	Chr.4: 73740952
rs4880	*SOD2*	C_8709053_10	Chr.6: 159692840
rs2297518	*NOS2*	C_11889257_10	Chr.17: 27769571
rs2779249		C___2593689_10	Chr.17: 27801555

**Fig 1:**
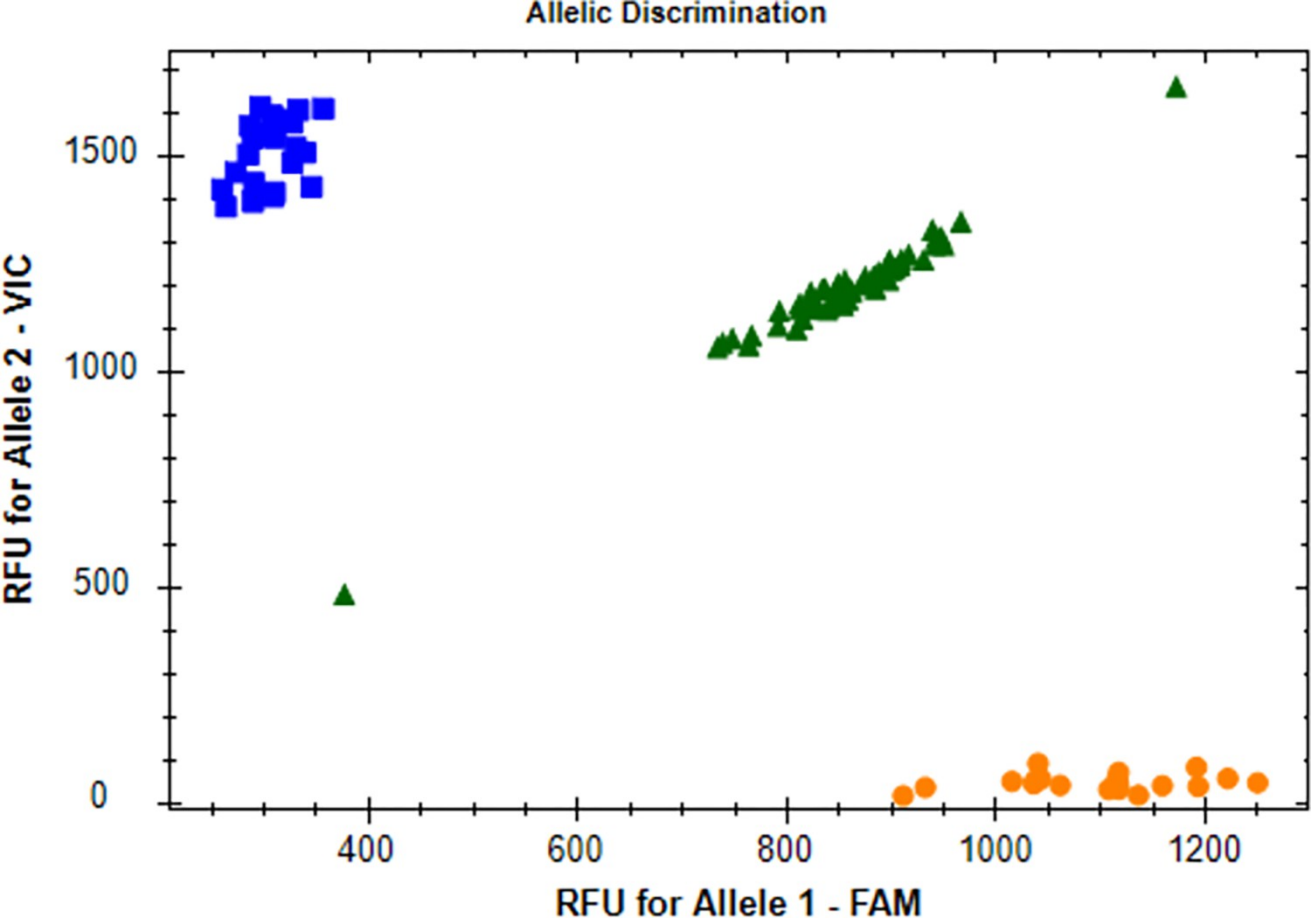
Genotype distribution of the rs2227307 polymorphism. Blue squares mean homozygous G/G, orange circles—homozygous T/T and green triangles—heterozygous T/G.

A representative allelic discrimination X–Y scatter-plot of the c.+396 T>G SNP (rs2227307) of the *IL-8* is presented in Fig 1.

### cDNA synthesis and mRNA expression levels

cDNA synthesis and mRNA expression levels were performed according to a protocol described by Grębowski et al. (2023) [27]. mRNA expression level was analysed by a TaqMan probe-based real-time PCR assay. The Taq-Man Expression Assay® (Thermo Fisher Scientific, Waltham, MA, USA) IDs and thermal cycling conditions are presented in Table 3. Finally, the relative transcript abundance of analysed genes was estimated by the 2^−ΔCt^ method, where ΔC_t_ sample  =  C_t target gene_ − C_t reference gene (18S)_ [34].

**Table 3:** Taq-man expression Assay® (Thermo Fisher Scientific, Waltham, MA, USA) and qPCR conditions.

Gene	Entrez Gene ID	RefSeq	TaqMan® Gene Expression Assay ID	qPCR conditions			
*18S* as reference gene	HSRRN18S	X03205.1 (GenBank mRNA)	Hs99999901_s1	**Step**	**Temperature**	**Time**	**Number of cycles**
*IL-6*	3569	NM_000600.4	Hs00174131_m1	AmpliTaq Gold Enzyme Activation	95⁰C	10 min	1
*IL-8*	3576	NM_000584.3	Hs00174103_m1	Denature	95⁰C	15 s	40
*SOD2*	6648	NM_000636.3	Hs00167309_m1	Anneal	60⁰C	60 s	
*NOS2*	4843	NM_000625.4	Hs01075529_m1				

### Statistical analysis

Statistica 12 (Statsoft, Tulsa, OK, USA) and SigmaPlot 11.0 (Systat Software Inc., San Jose, CA, USA) programs were used for statistical analysis. χ^2^ test was used to Hardy–Weinberg equilibrium of the observed genotype frequencies with the expected frequencies among the case and control subjects. The χ^2^ analysis was also used to investigate the significance of differences between genotype and allele distributions in studied groups. The unconditional multiple logistic regression model (codominant, dominant, and recessive models) was used to obtain the ORs and its corresponding 95% CI with p-values for urolithiasis risk. Additionally, the OR was adjusted for gender, as men are exposed to a higher risk of urolithiasis development in comparison to women [5]. Moreover, the SF analysis proposed by Mario Cortina-Borja et al. (2009) was used for the assessment of the potential SNP-SNP interactions and associations with urolithiasis [35]. Linkage disequilibrium (LD) and haplotype distribution were assessed based on known genotypes of four SNPs (rs1800797 and rs2069845; rs2297518 and rs2779249) and the SHEsisPlus software (http://shesisplus.bio-x.cn/SHEsis.html, accessed on 28 November 2022) [36] was used. In the case of LD analysis, haplotypes with a frequency less than 0.03 were excluded from the LD analysis. We also evaluated the association between the cases and controls for each studied polymorphism in the male/female population or non-smoker/smoker groups or subpopulations with the normal body weight/overweight/obesity group by using the unconditional logistic regression model. The data presenting the results from the distribution of genotypes in terms of the age of the first renal colic attack and the number of the attacks are shown as median with an interquartile range. Distribution normality was examined using the Shapiro–Wilk test, and then, the significance of the difference between studied values was determined based on the Mann–Whitney test or Student’s t-test. Demographics and baseline characteristics of patients were compared by unpaired Student’s t-test or χ^2^ test, as appropriate, and baseline relative mRNA expressions were compared by the Mann-Whitney test. Moreover, the Kruskal-Wallis One Way Analysis of Variance on Ranks was used to compare relative mRNA expression between groups concerning genotype. Moreover, data regarding the effects of gender/BMI and urolithiasis on mRNA expression of *IL-6*, *IL-8*, *SOD2*, and *NOS2* were analysed using two-way ANOVA analyses. Finally, the Bonferroni test was used as a post-hoc test. The values of *p* < 0.05 were considered statistically significant.

## Results

### Patients

112 patients with urolithiasis and 114 controls were recruited in our study. The detailed characteristics of the study participants obtained from the structured questionnaire designed for earlier studies [27] are presented in Table 4. In addition, the structural characteristics of the study groups were supplemented with clinical data, which were selected based on previously performed studies [27]. The number of males was higher than women in both groups. Moreover, the study participants recruited into the control group are older than the patients diagnosed with urolithiasis (*p* < 0.001). The higher age in the control group was to help avoid qualifying young people who may suffer from urolithiasis in the future, despite the lack of confirmation of this disease in the family history. Due to the higher average age, the control group is dominated by people who are economically inactive (retired), while among patients with urolithiasis, there is a significant number of people working physically compared to the controls (*p* < 0.001). An analysis of the total blood count of all study participants showed that urolithiasis patients were characterised by a decreased level of mean corpuscular volume as compared to controls (*p* < 0.01). Moreover, the mean corpuscular haemoglobin concentration, the level of white blood cells, and the level of blood platelets were higher in patients with urolithiasis than in healthy volunteers (*p* < 0.01). In the case of blood biochemical parameters, statistical analysis showed that patients with urolithiasis showed higher levels of glucose (*p* < 0.01) and creatinine (*p* < 0.001) than controls. In turn, dipstick urinalysis confirmed that urine pH was lower (acidic) in patients with urolithiasis compared to the control group (*p* < 0.05). Moreover, the urine of urolithiasis patients was characterised by higher levels of protein (*p* < 0.001) and ketones (*p* < 0.01). Moreover, an analysis of urine microscopy confirmed that the urine of patients contained more red blood cells (*p* < 0.001) and bacteria (*p* < 0.001).

**Table 4:** Clinical-demographic characteristics of patients with urolithiasis and control based on a questionnaire developed for a previous study [27].

**Demographic characteristics of the study participants**					
Feature			Controls (n = 114) Frequency	Patients with urolithiasis (n = 112) Frequency	*p* *
Gender	females		0.34	0.32	0.743
	males		0.66	0.68	
**Age**	**mean ± SD**		**66.71 ± 11.76**	**56.31 ± 14.36**	**<0.001**
	**range**		**28–91**	**26–80**	
Education	primary (basic) education		0.16	0.15	0.096
	vocational education		0.39	0.27	
	high school education		0.33	0.39	
	university degree		0.12	0.19	
Residency	village		0.36	0.45	0.529
	a city with a population under 50 thou. residents		0.39	0.28	
	a city with a population over 50 thou. residents		0.25	0.28	
Marital status	single		0.25	0.13	0.152
	married		0.67	0.82	
	widow/widower		0.08	0.05	
**Professional activity**	**physical work**		**0.28**	**0.40**	**<0.001**
	**mental work**		**0.17**	**0.21**	
	**unemployment**		**0.01**	**0.05**	
	**pension**		**0.64**	**0.40**	
Smoking	never		0.56	0.51	0.066
	former		0.24	0.23	
	current		0.20	0.26	
BMI [kg/m^2]^	mean ± SD		27.69 ± 3.51	28.28 ± 5.00	0.424
	< 25		0.31	0.30	
	25–30		0.42	0.37	
	>30		0.27	0.33	
Daily fluid intake	< 2 L/day		0.52	0.54	0.685
	> 2 L/day		0.48	0.46	
Daily coffee consumption (number of cups, one cup has a capacity of 200 mL)	0		0.21	0.27	0.209
	1		0.44	0.46	
	2–3		0.32	0.22	
	> 4		0.03	0.05	
**Total blood count of the study participants**					
Feature/Parameters			Controls (n = 114) mean ± SD	Patients with urolithiasis (n = 112) mean ± SD	*p*
Red Blood Cells–RBC (x10^12^/L)			4.49 ± 0.61	4.60 ± 0.56	0.161
Haematocrit–HCT (%)			40.91 ± 5.26	41.21 ± 4.79	0.674
Haemoglobin–HGB (g/L)			13.56 ± 2.44	14.01 ± 1.76	0.104
**Mean corpuscular volume–MCV (fL)**			**91.25 ± 5.34**	**89.75 ± 5.40**	**0.004**
Mean cell haemoglobin–MCH (pg/cell)			30.20 ± 3.20	30.47 ± 1.89	0.791
**Mean corpuscular haemoglobin concentration–MCHC (g/L)**			**33.27 ± 1.22**	**33.98 ± 0.86**	**<0.001**
Red cell distribution width–RDW (%)			13.50 ± 1.49	13.31 ± 1.10	0.216
Haemoglobin distribution width–HDW (g/L)			2.53 ± 0.25	2.52 ± 0.27	0.624
**White blood cells–WBC (x10** ^**9**^ **/L)**			**7.47 ± 2.35**	**9.94 ± 4.05**	**<0.001**
%HYPO			2.34 ± 2.23	1.27 ± 1.21	0.267
%MIKRO			1.35 ± 1.26	0.91 ± 0.84	0.704
%MAKRO			1.44 ± 1.15	1.22 ± 1.20	0.817
%HYPER			0.73 ± 0.54	0.91 ± 0.68	0.069
**Blood platelets–PLT (x10** ^**9**^ **/L)**			**234.95 ± 70.80**	**269.30 ± 103.43**	**0.008**
Mean platelet volume–MPV (fL)			8.68 ± 1.53	8.79 ± 1.31	0.391
**Blood biochemical parameters**					
Feature/Parameters			Controls (n = 114) mean ± SD	Patients with urolithiasis (n = 112) mean ± SD	*p*
**Glucose (mmol/L)**			**5.92 ± 2.16**	**6.44 ± 1.97**	**0.009**
**Creatinine (μmol/L)**			**106.94 ± 12.14**	**119.28 ± 9.11**	**<0.001**
Sodium (mmol/L)			139.93 ± 2.75	139.76 ± 2.80	0.654
Potassium (mmol/L)			5.57 ± 9.42	4.38 ± 0.45	0.282
**Coagulation panel**					
Prothrombin time (s)			12.68 ± 2.48	12.30 ± 1.98	0.174
Prothrombin index (%)			92.90 ± 11.89	95.01 ± 10.52	0.176
International normalized ratio (INR)			1.13 ± 0.23	1.11 ± 0.18	0.883
Activated partial thromboplastin time (APTT, s)			30.67 ± 3.91	29.78 ± 2.76	0.201
Fibrinogen (mg/L)			400.24 ± 145.26	456.39 ± 194.31	0.134
**Dipstick urinalysis**					
Feature/Parameters			Controls (n = 114) mean ± SD	Patients with urolithiasis (n = 112) mean ± SD	*p*
**pH**			**5.84 ± 0.88**	**5.63 ± 0.84**	**0.039**
Specific gravity			1.02 ± 0.01	1.02 ± 0.01	0.999
Feature/Parameters			Controls (n = 114) Frequency	Patients with urolithiasis (n = 112) Frequency	*p*
WBC	absence per high-power field		0.68	0.54	0.060
	single per high-power field		0.29	0.46	
	numerous per high-power field		0.04	0.00	
Nitrite	negative		0.88	0.91	0.416
	positive		0.12	0.09	
Glucose	negative		0.98	0.94	0.085
	positive		0.02	0.06	
**Protein**	**negative**		**0.76**	**0.55**	**<0.001**
	**positive**		**0.24**	**0.45**	
**Ketones**	**negative**		**0.90**	**0.78**	**0.009**
	**positive**		**0.10**	**0.22**	
Bilirubin	negative		0.98	0.96	0.399
	positive		0.02	0.04	
Urobilinogen	normal level		1.00	0.97	0.080
	above normal		0.00	0.03	
Colour	pale yellow		0.05	0.13	0.328
	straw/yellow		0.82	0.62	
	dark yellow		0.02	0.01	
	amber		0.05	0.21	
	brown		0.01	0.03	
	red		0.04	0.02	
Clarity	clear		0.74	0.63	0.183
	slightly cloudy		0.12	0.22	
	cloudy		0.06	0.10	
	very cloudy		0.08	0.04	
**Urine microscopy**					
Feature/Parameters			Controls (n = 114) Frequency	Patients with urolithiasis (n = 112) Frequency	*p*
**RBC**	**0-3/high power field**		**0.78**	**0.42**	**<0.001**
	**3-5/high power field**		**0.07**	**0.13**	
	**5-10//high power field**		**0.03**	**0.19**	
	**10-15/high power field**		**0.03**	**0.06**	
	**15-20/high power field**		**0.03**	**0.09**	
	**20-25/high power field**		**0.06**	**0.12**	
WBC	1-3/high power field		0.66	0.58	0.256
	3-5/high power field		0.05	0.08	
	5-10/high power field		0.19	0.20	
	10-15/high power field		0.01	0.05	
	20-25/high power field		0.08	0.08	
Squamous epithelial cells	single per high power field		0.82	0.81	0.173
	sparse per high power field		0.10	0.14	
	many per high power field		0.08	0.05	
Mucus thread	single per high power field		0.00	0.17	0.378
	sparse per high power field		0.80	0.40	
	many per high power field		0.20	0.43	
**Bacteria**	**single per high power field**		**0.65**	**0.01**	**<0.001**
	**sparse per high power field**		**0.27**	**0.48**	
	**many per high power field**		**0.08**	**0.51**	
**Additional information**					
Feature/Parameters			Controls (n = 114) Frequency	Patients with urolithiasis (n = 112) Frequency	*p*
Comorbidities	hypertension	yes	0.53	0.53	0.995
		no	0.47	0.47	
	diabetes	yes	0.17	0.21	0.362
		no	0.83	0.79	
	**hypercholesterolaemia**	**yes**	**0.22**	**0.12**	**0.039**
		**no**	**0.78**	**0.88**	
Number of attacks of renal colic	1		n/d	0.39	n/d
	≥ 2		n/d	0.61	
Location of the urinary stone	Urinary bladder		n/d	0.46	n/d
	Ureters		n/d	0.43	
	Kidneys		n/d	0.11	
Previous therapy in the case of recurrence of urolithiasis	Ureteroscopic lithotripsy (URSL)		n/d	0.23	n/d
	Retrograde intrarenal surgery (RIRS)		n/d	0.02	
	Percutaneous nephrolithotomy (PCNL)		n/d	0.01	
	Extracorporeal shock wave lithotripsy (ESWL)		n/d	0.09	
	Chirurgical therapy		n/d	0.06	
	DJ catheter		n/d	0.18	
	Conservative treatment (oral medication, injections, drips)		n/d	0.26	
	Spontaneous removal of deposits		n/d	0.14	

*p < 0.05 are in bold

### Single nucleotide polymorphisms of the inflammation as well as oxidative and nitrative stress-related genes as the risk of urolithiasis occurrence

As shown in Table 5, only *SOD2* polymorphism was associated with the change in the frequency of urolithiasis. We observed that the C/T genotype of the c.47 T>C–*SOD2* SNP was linked with an increased frequency of urolithiasis occurrence (OR 1.726; 1.016–2.993; 95% CI; *p* < 0.05) whereas the T/T homozygote of the same polymorphism decreased the risk of urolithiasis development (OR 0.489; 0.248–0.961; 95% CI; *p* < 0.05) in the Polish population. Moreover, in the case of the left-over analysed SNPs, our study showed (Table 5) no differences between urolithiasis patients and healthy volunteers in terms of the distribution of genotypes.

**Table 5:** Distribution of genotypes and alleles of the 597 A>G–*IL-6* (rs1800797) and c.3331 G>A–*IL-6* (rs2069845), c.+396 T>G–*IL-8* (rs2227307), c.47 T>C (p.Val16Ala)–*SOD2* (rs4880), c.1823 C>T (p.Ser608Leu)–*NOS2* (rs2297518) and g.-1026 C>A–*NOS2* (rs2779249) and ORs with 95% CIs in patients with urolithiasis and controls.

Genotypes/Alleles	Control (n = 114)		Urolithiasis (n = 112)		Crude OR (95% CI)*	*p*	Adjusted OR (95% CI)*	*p* *
	Number	Frequency	Number	Frequency				
**-597 A>G–*IL-6* (rs1800797)**								
Frequencies								
A/A	23	0.202	28	0.250	1.319 (0.705–2.467)	0.386	1.316 (0.704–2.463)	0.390
A/G	58	0.509	57	0.509	1.001 (0.594–1.686)	0.998	0.999 (0.593–1.683)	0.996
G/G	33	0.289	27	0.241	0.780 (0.431–1.410)	0.411	0.783 (0.432–1.417)	0.418
χ^2^ = 226.00; p = 0.431								
A	104	0.456	113	0.504	1.219 (0.838–1.773)	0.300	1.216 (0.836–1.770)	0.306
G	124	0.544	111	0.496	0.820 (0.564–1.193)	0.300	0.822 (0.565–1.196)	0.306
Carriage rates								
A (+)	81	0.711	85	0.759	1.283 (0.709–2.320)	0.411	1.278 (0.706–2.313)	0.418
A (-)	33	0.289	27	0.241	0.780 (0.431–1.410)	0.411	0.783 (0.432–1.417)	0.418
G (+)	91	0.798	84	0.750	0.758 (0.405–1.418)	0.386	0.760 (0.406–1.421)	0.390
G (-)	23	0.202	28	0.250	1.319 (0.705–2.467)	0.386	1.316 (0.704–2.463)	0.390
**c.3331 G>A–*IL-6* (rs2069845)**								
Frequencies								
G/G	26	0.228	29	0.259	1.183 (0.644–2.173)	0.589	1.179 (0.642–2.168)	0.595
G/A	56	0.491	59	0.527	1.153 (0.684–1.943)	0.593	1.153 (0.684–1.943)	0.594
A/A	32	0.281	24	0.214	0.699 (0.380–1.285)	0.249	0.701 (0.381–1.289)	0.253
χ^2^ = 225.999; p = 0.431								
G	108	0.474	117	0.522	1.220 (0.839–1.773)	0.298	1.217 (0.837–1.770)	0.303
A	120	0.526	107	0.478	0.820 (0.564–1.191)	0.298	0.821 (0.565–1.194)	0.303
Carriage rates								
G (+)	82	0.719	88	0.786	1.431 (0.778–2.630)	0.249	1.427 (0.776–2.624)	0.253
G (-)	32	0.281	24	0.214	0.699 (0.380–1.285)	0.249	0.701 (0.381–1.289)	0.253
A (+)	88	0.772	83	0.741	0.846 (0.460–1.554)	0.589	0.848 (0.461–1.558)	0.595
A (-)	26	0.228	29	0.259	1.183 (0.644–2.173)	0.589	1.179 (0.642–2.168)	0.595
**c.+396 T>G–*IL-8* (rs2227307)**								
Frequencies								
T/T	27	0.237	28	0.250	1.074 (0.585–1.972)	0.818	1.071 (0.583–1.968)	0.825
T/G	61	0.535	59	0.527	0.967 (0.574–1.631)	0.900	0.969 (0.575–1.635)	0.906
G/G	26	0.228	25	0.223	0.973 (0.521–1.815)	0.930	0.973 (0.521–1.816)	0.931
χ^2^ = 226.000; p = 0.431								
T	115	0.504	115	0.513	1.039 (0.710–1.521)	0.843	1.038 (0.709–1.520)	0.848
G	113	0.496	109	0.487	0.962 (0.657–1.408)	0.843	0.963 (0.658–1.410)	0.848
Carriage rates								
T (+)	88	0.772	87	0.777	1.028 (0.551–1.919)	0.930	1.028 (0.551–1.919)	0.931
T (-)	26	0.228	25	0.223	0.973 (0.521–1.815)	0.930	0.973 (0.521–1.816)	0.931
G (+)	87	0.763	84	0.750	0.931 (0.507–1.710)	0.818	0.934 (0.508–1.715)	0.825
G (-)	27	0.237	28	0.250	1.074 (0.585–1.972)	0.818	1.071 (0.583–1.968)	0.825
**c. 47 C>T (p.Val16Ala)–*SOD2* (rs4880)**								
Frequencies								
C/C	29	0.254	26	0.232	0.886 (0.482–1.628)	0.697	0.884 (0.481–1.624)	0.690
**C/T**	**56**	**0.491**	**70**	**0.625**	**1.726 (1.016–2.933)**	**0.044**	**1.722 (1.013–2.928)**	**0.045**
**T/T**	**29**	**0.254**	**16**	**0.143**	**0.489 (0.248–0.961)**	**0.038**	**0.491 (0.249–0.967)**	**0.040**
χ^2^ = 225.991; p = 0.432								
C	114	0.500	102	0.455	1.226 (0.826–1.819)	0.313	1.222 (0.822–1.814)	0.321
T	114	0.500	122	0.545	0.816 (0.550–1.211)	0.313	0.819 (0.551–1.216)	0.321
Carriage rates								
**C (+)**	**85**	**0.746**	**86**	**0.768**	**2.047 (1.041–4.027)**	**0.038**	**2.038 (1.035–4.016)**	**0.040**
**C (-)**	**29**	**0.254**	**16**	**0.143**	**0.489 (0.248–0.961)**	**0.038**	**0.491 (0.249–0.967)**	**0.040**
T (+)	85	0.746	96	0.857	1.129 (0.614–2.073)	0.697	1.132 (0.616–2.080)	0.690
T (-)	29	0.254	26	0.232	0.886 (0.482–1.628)	0.697	0.884 (0.481–1.624)	0.690
**c.1823 C>T (p. Ser608Leu)–*NOS2* (rs2297518)**								
Frequencies								
C/C	76	0.667	78	0.696	1.147 (0.655–2.009)	0.631	1.138 (0.648–1.998)	0.654
C/T	35	0.307	30	0.268	0.826 (0.464–1.471)	0.516	0.834 (0.466–1.493)	0.541
T/T	3	0.026	4	0.036	1.370 (0.300–6.267)	0.685	1.348 (0.294–6.188)	0.701
χ^2^ = 226.000; p = 0.431								
C	187	0.820	186	0.830	1.073 (0.660–1.743)	0.776	1.068 (0.656–1.736)	0.792
T	41	0.180	38	0.170	0.932 (0.574–1.514)	0.776	0.937 (0.576–1.523)	0.792
Carriage rates								
C (+)	111	0.974	108	0.964	0.730 (0.160–3.337)	0.685	0.742 (0.162–3.407)	0.701
C (-)	3	0.026	4	0.036	1.370 (0.300–6.267)	0.685	1.348 (0.294–6.118)	0.701
T (+)	38	0.333	34	0.303	0.872 (0.498–1.527)	0.631	0.879 (0.500–1.544)	0.654
T (-)	76	0.667	78	0.697	1.147 (0.655–2.009)	0.631	1.138 (0.648–1.998)	0.654
**g.-1026 C>A–*NOS2* (rs2779249)**								
Frequencies								
C/C	57	0.500	57	0.509	1.036 (0.615–1.746)	0.893	1.028 (0.609–1.736)	0.917
C/A	51	0.447	47	0.420	0.893 (0.528–1.512)	0.674	0.899 (0.530–1.515)	0.694
A/A	6	0.053	8	0.071	1.385 (0.465–4.127)	0.559	1.388 (0.466–4.140)	0.556
χ^2^ = 226.000; p = 0.431								
C	165	0.724	161	0.719	0.974 (0.634–1.494)	0.903	0.968 (0.630–1.488)	0.882
A	63	0.276	63	0.281	1.027 (0.669–1.576)	0.903	1.033 (0.672–1.588)	0.882
Carriage rates								
C (+)	108	0.947	104	0.929	0.722 (0.242–2.153)	0.559	0.720 (0.242–2.148)	0.556
C (-)	6	0.053	8	0.071	1.385 (0.465–4.127)	0.559	1.388 (0.466–4.140)	0.556
A (+)	57	0.500	55	0.491	0.965 (0.573–1.626)	0.893	0.972 (0.576–1.642)	0.917
A (-)	57	0.500	57	0.509	1.036 (0.615–1.746)	0.893	1.028 (0.609–1.736)	0.917

*p < 0.05 along with the corresponding ORs are in bold for the genotypes/alleles increasing the risk of urolithiasis and for the genotypes/alleles with a protective effect).

### Association between combined genotypes of *IL-6*, *IL-8*, *SOD2* and *NOS2* polymorphisms and the risk of urolithiasis occurrence–gene-gene interaction

We also studied the link between urolithiasis occurrence and combined genotypes of the tested polymorphisms localised in genes, encoding interleukins as well as enzymes associated with both oxidative and nitrative stress. These results are presented in Table 6. We found that A/A-C/T combined genotypes of -597 A>G (rs1800797)–*IL-6* and c. 47 C>T–*SOD2* (rs4880) polymorphisms were associated with an evaluated risk of urolithiasis development (Crude OR 2.537; 1.055–6.104 95% CI; *p* < 0.05). Furthermore, in the case of c. 47 C>T–*SOD2* (rs4880) and c.3331 G>A–*IL-6* (rs2069845) the C/T-G/G combined genotypes were associated with an increased occurrence of urolithiasis (OR 2.707; 1.132–6.473 95% CI; *p* < 0.05). Moreover, in the other studied combined polymorphisms, we detected no statistical significance (S1 Table).

**Table 6:** Gene-gene interactions of studied inflammation and oxidative stress-related polymorphisms and urolithiasis risk.

Combined genotypes	Control (n = 114)		Urolithiasis (n = 112)		Crude OR (95% CI)*	*p*	Adjusted OR (95% CI)*	*p* *
	Number	Frequency	Number	Frequency				
**-597 A>G (rs1800797)–*IL-6* and c. 47C>T–*SOD2* (rs4880)**								
A/A-T/T	7	0.061	6	0.054	0.865 (0.281–2.660)	0.801	0.870 (0.283–2.675)	0.807
**A/A-C/T**	**8**	**0.070**	**18**	**0.161**	**2.537 (1.055–6.104)**	**0.038**	**2.532 (1.052–6.094)**	**0.038**
A/A-C/C	8	0.070	4	0.036	0.491 (0.143–1.679)	0.257	0.486 (0.142–1.665)	0.251
A/G-T/T	13	0.114	8	0.071	0.598 (0.238–1.503)	0.274	0.594 (0.236–1.496)	0.269
A/G-C/T	32	0.281	34	0.304	1.117 (0.629–1.982)	0.705	1.117 (0.629–1.983)	0.705
A/G-C/C	13	0.114	15	0.134	1.201 (0.543–2.656)	0.650	1.200 (0.543–2.654)	0.652
G/G/T/T	9	0.079	2	0.018	0.212 (0.045–1.005)	0.051	0.212 (0.044–1.017)	0.053
G/G-C/T	16	0.140	18	0.161	1.173 (0.565–2.435)	0.669	1.168 (0.562–2.427)	0.677
G/G-C/C	8	0.070	7	0.063	0.883 (0.309–2.523)	0.817	0.883 (0.309–2.524)	0.817
**c. 47 C>T–*SOD2* (rs4880) and c.3331 G>A–*IL-6* (rs2069845)**								
T/T-G/G	9	0.079	5	0.045	0.545 (0.177–1.681)	0.291	1.081 (0.620–1.886)	0.298
T/T-G/A	11	0.096	9	0.080	0.818 (0.325–1.058)	0.670	0.811 (0.322–2.042)	0.656
T/T- A/A	9	0.079	2	0.018	0.212 (0.045–1.005)	0.051	0.212 (0.044–1.017)	0.053
**C/T-G/G**	**8**	**0.070**	**19**	**0.170**	**2.707 (1.132–6.473)**	**0.025**	**2.699 (1.129–6.456)**	**0.026**
C/T-G/A	33	0.289	35	0.313	1.116 (0.632–1.970)	0.706	1.119 (0.633–1.977)	0.699
C/T-A/A	15	0.132	16	0.143	1.100 (0.515–2.348)	0.805	1.092 (0.511–2.334)	0.821
C/C-G/G	9	0.079	5	0.045	0.545 (0.177–1.681)	0.291	0.537 (0.174–1.661)	0.281
C/C-G/A	12	0.105	15	0.134	1.314 (0.586–2.950)	0.507	1.315 (0.586–2.951)	0.507
C/C-A/A	8	0.070	6	0.054	0.750 (0.252–2.236)	0.606	0.753 (0.252–2.242)	0.609

*p < 0.05 along with the corresponding ORs are in bold (for the genotypes/alleles increasing the risk of urolithiasis)

However, synergy factor (SF) analysis (Table 7) proposed by Mario Cortina-Borja et al. (2009) [35] showed that the antagonistic interaction was only seen between c.+396 T>G–*IL-8* (rs2227307) and c.3331 G>A–*IL-6* (rs2069845) (SF  =  4.617, *p* < 0.05).

**Table 7:** Synergy factor analysis.

Genes	Polymorphism	Subjects	Synergy factor ^a^	*p-*value ^b^	Type interaction ^a^
*IL-6 × IL-8*	-597 A>G (rs1800797) × c.+396 T>G (rs2227307)	G carriers–G carriers	3.273	0.122	Antagonistic
*IL-6 × NOS2*	-597 A>G (rs1800797) × c.1823 C>T (p. Ser608Leu) (rs2297518)	G carriers–T carriers	0.949	0.940	Synergistic
*IL-6 × NOS2*	-597 A>G (rs1800797) × g.-1026 C>A (rs2779249)	G carriers–A carriers	1.524	0.513	Antagonistic
*SOD2 × NOS2*	c.47 C>T (p.Val16Ala) (rs4880) × c.1823 C>T (p. Ser608Leu) (rs2297518)	T carriers–T carriers	0.847	0.805	Synergistic
*SOD2 × NOS2*	c.47 C>T (p.Val16Ala) (rs4880) × g.-1026 C>A (rs2779249)	T carriers–A carriers	1.567	0.472	Antagonistic
*SOD2 × IL-8*	c.47 C>T (p.Val16Ala) (rs4880) × c.+396 T>G (rs2227307)	T carriers–G carriers	2.348	0.232	Antagonistic
*Il-8 × NOS2*	c.+396 T>G (rs2227307) × g.-1026 C>A (rs2779249)	G carriers–A carriers	1.051	0.936	Antagonistic
*Il-8 × NOS2*	c.+396 T>G (rs2227307) × c.1823 C>T (p. Ser608Leu) (rs2297518)	G carriers–T carriers	0.716	0.618	Synergistic
***IL-8 × IL-6***	**c.+396 T>G (rs2227307) × c.3331 G>A (rs2069845)**	**G carriers–A carriers**	**4.617**	**0.044**	**Antagonistic**
*IL-6 × NOS2*	c.3331 G>A (rs2069845) × g.-1026 C>A (rs2779249)	A carriers–A carriers	2.254	0.196	Antagonistic
*IL-6 × NOS2*	c.3331 G>A (rs2069845) × c.1823 C>T (p. Ser608Leu) (rs2297518)	A carriers–T carriers	1.604	0.478	Antagonistic
*IL-6 × SOD2*	-597 A>G (rs1800797) × c.47 C>T (p.Val16Ala) (rs4880)	G carriers–T carriers	0.264	0.088	Antagonistic
*SOD2 × IL-6*	c.47 C>T (p.Val16Ala) (rs4880) × c.3331 G>A (rs2069845)	T carriers–A carriers	0.342	0.144	Antagonistic

*p* < 0.05 are bold for the genotypes with an increased risk effect.

^a^ All SF relate to the risk of urolithiasis. The cited genotypes were treated as risk factors unless otherwise stated; the terms, ‘risk’ and ‘protective’ factors, refer to associations, i.e. no causality is implied. Note that synergy (antagonism) between risk factors will produce an SF > 1 (< 1), while synergy (antagonism) between protective factors will give an SF < 1 (> 1).

^b^ All *p*-values are before correction for multiple testing, whether or not relevant.

### Linkage disequilibrium and haplotype analysis

**Fig 2:**
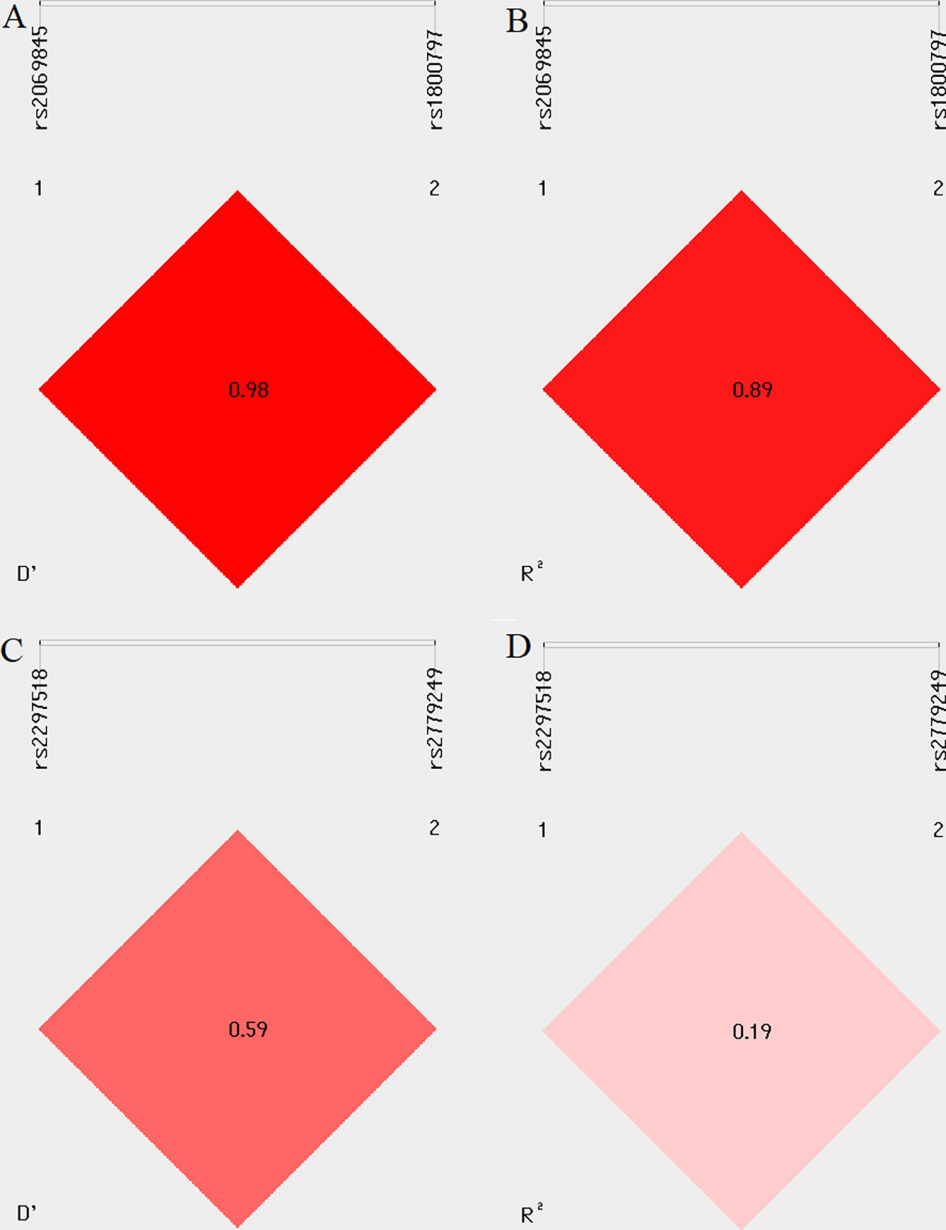
Analysis of LD of rs2069845 and rs1800797 polymorphisms in the *IL-6* gene (A, B) as well as rs2297518 and 2779249 polymorphisms in the *NOS2* gene (C, D). Pairwise D’ values (A, C). Pairwise R^2^ values (B, D). R^2^ ≥ 0.8 –high LD.

LD analysis revealed that among studied SNPs in the *IL-6* and *NOS2* genes, we identified rs2069845 and rs1800797 polymorphisms as a strong linkage disequilibrium region in *IL-6* (R^2^ ≥ 0.8) (Fig 2). The analysis of the haplotypes of the examined SNPs associated with urolithiasis occurrence and the results are presented in S2 Table. Unfortunately, none of the studied haplotypes were significantly associated with urolithiasis in the case of *IL-6* and *NOS2* genes.

### SNPs of genes encoding interleukins as well as enzymes involved in nitrative and oxidative stresses and urolithiasis occurrence in the male and female subpopulation

Despite the increase in the number of new cases of urolithiasis in the group of women, current epidemiological data still indicate that men suffer from the disease more often than women [5]. Therefore, we analysed the link between the occurrence of urolithiasis in male or female groups and all studied polymorphic variants.

Interestingly, our findings confirmed that the SNPs may modulate the risk of urolithiasis development depending on the gender of the patient (Table 8 and S3 Table). We found that the heterozygote of g.-1026 C>A–*NOS2* (rs2779249) polymorphism was associated with a reduced risk of urolithiasis development in the female subpopulation (Crude OR 0.353; 0.138–0.902 95% CI; *p* < 0.05), while in the male subpopulation, we did not observe this association.

**Table 8:** Distribution of genotypes and alleles of the g.-1026 C>A–*NOS2* (rs2779249) and ORs with 95% CIs in men and women with urolithiasis.

Genotypes/Alleles	WOMEN (n = 75)				MEN (n = 151)			
	Control (n = 39)	Urolithiasis (n = 36)	Crude OR (95% CI)*	*p*	Control (n = 75)	Urolithiasis (n = 76)	Crude OR (95% CI)*	*p* *
	N (Freq.)	N (Freq.)			N (Freq.)	N (Freq.)		
**g.-1026 C>A–*NOS2* (rs2779249)**								
C/C	13 (0.333)	20 (0.556)	2.500 (0.981–6.372)	0.055	44 (0.587)	37 (0.487)	0.668 (0.351–1.272)	0.220
**C/A**	**24 (0.615)**	**13 (0.361)**	**0.353 (0.138–0.902)**	**0.030**	27 (0.360)	34 (0.447)	1.439 (0.749–2.766)	0.275
A/A	2 (0.051)	3 (0.083)	1.682 (0.265–10.693)	0.582	4 (0.053)	5 (0.066)	1.250 (0.322–4.847)	0.747
χ^2^ = 75.238; *p =* 0.374					χ^2^ = 151.016; *p =* 0.416			
C	50 (0.641)	53 (0.736)	1.698 (0.785–3.670)	0.179	115 (0.767)	108 (0.711)	0.736 (0.432–1.252	0.258
A	28 (0.359)	19 (0.264)	0.589 (0.272–1.274)	0.179	35 (0.233)	44 (0.289)	1.359 (0.799–2.312)	0.258

*p < 0.05 along with the corresponding ORs are in bold for the genotypes/alleles decreasing the risk of urolithiasis

### SNPs of genes encoding interleukins as well as enzymes involved in nitrative and oxidative stresses and urolithiasis occurrence in non-smoker/smoker subpopulation and group with normal body weight/overweight and obesity

Previous epidemiological data among the risk factors favouring the development and recurrence of urolithiasis indicate cigarette smoking, as well as being overweight and obese [12–17]. Thus, we analysed the interdependence between all examined SNPs and the urolithiasis occurrence in non-smoker or smoker groups (Table 9 and S4 Table) as well as subjects with normal body weight or subjects with overweight and obesity (BMI≥25, Table 10 and S5 Table).

**Table 9:** Distribution of genotypes and alleles of the c.47 T>C (p.Val16Ala)–*SOD2* (rs4880) and ORs with 95% CIs in non-smokers and smokers.

Genotypes/Alleles	NON-SMOKER (n = 127)				SMOKER (n = 99)			
	Control (n = 68)	Urolithiasis (n = 59)	Crude OR (95% CI)*	*p*	Control (n = 46)	Urolithiasis (n = 53)	Crude OR (95% CI)*	*p* *
	N (Freq.)	N (Freq.)			N (Freq.)	N (Freq.)		
**c.47 T>C (p.Val16Ala)–*SOD2* (rs4880)**								
C/C	17 (0.250)	14 (0.237)	0.933 (0.414–2.104)	0.868	12 (0.261)	12 (0.226)	0.829 (0.330–2.081)	0.690
**T/C**	35 (0.515)	34 (0.576)	1.282 (0.635–2.587)	0.488	**21 (0.457)**	**36 (0.679)**	**2.521 (1.112–5.713)**	**0.027**
**T/T**	16 (0.235)	11 (0.186)	0.745 (0.315–1.764)	0.503	**13 (0.283)**	**5 (0.094)**	**0.264 (0.086–0.813)**	**0.020**
χ^2^ = 126.998; *p =* 0.409					χ^2^ = 99.097; *p =* 0.394			
C	69 (0.507)	62 (0.525)	1.083 (0.646–1.816)	0.764	45 (0.489)	60 (0.566)	1.447 (0.779–2.688)	0.242
T	67 (0.493)	56 (0.475)	0.924 (0.551–1.549)	0.764	47 (0.511)	46 (0.434)	0.691 (0.372–1.283)	0.242

*p < 0.05 along with the corresponding ORs are in bold (for the genotypes/alleles increasing the risk of urolithiasis and for the genotypes/alleles with a protective effect).

**Table 10:** Distribution of genotypes and alleles of the c.47 T>C (p.Val16Ala)–*SOD2* (rs4880) and ORs with 95% CIs in subjects with normal body weight or subjects with overweight and obesity.

Genotypes/Alleles	BMI < 25 (n = 75)				BMI ≥ 25 (n = 151)			
	Control (n = 39)	Urolithiasis (n = 36)	Crude OR (95% CI)*	*p*	Control (n = 75)	Urolithiasis (n = 76)	Crude OR (95% CI)*	*p* *
	N (Freq.)	N (Freq.)			N (Freq.)	N (Freq.)		
**c.47 T>C (p.Val16Ala)–*SOD2* (rs4880)**								
C/C	10 (0.256)	8 (0.222)	0.826 (0.286–2.403)	0.729	19 (0.253)	18 (0.237)	0.915 (0.436–1.921)	0.814
**T/C**	21 (0.538)	22 (0.611)	1.347 (0.537–3.378)	0.525	**35 (0.467)**	**48 (0.632)**	**1.959 (1.022–3.754)**	**0.043**
**T/T**	8 (0.205)	6 (0.167)	0.775 (0.240–2.501)	0.670	**21 (0.280)**	**10 (0.132)**	**0.390 (0.169–0.898)**	**0.027**
χ^2^ = 75.000; *p =* 0.381					χ^2^ = 151.001; *p =* 0.416			
C	41 (0.526)	38 (0.528)	1.010 (0.504–2.026)	0.977	73 (0.487)	84 (0.553)	1.345 (0.831–2.176)	0.228
T	37 (0.474)	34 (0.472)	0.990 (0.494–1.985)	0.977	77 (0.513)	68 (0.447)	0.744 (0.460–1.204)	0.228

*p < 0.05 along with the corresponding ORs are in bold (for the genotypes/alleles increasing the risk of urolithiasis and for the genotypes/alleles with a protective effect).

Interestingly, our findings showed that in the smoker group, the T/C genotype of c.47 T>C (p.Val16Ala)–*SOD2* (rs4880) SNP was associated with an elevated risk of urolithiasis development (Crude OR 2.521; 1.112–5.713 95% CI; *p* < 0.05), whereas in non-smoker group we did not observe this association. On the other hand, the C/C homozygote of the same polymorphism was linked with a reduction in this risk only in the group of smokers.

Similarly, in the group of subjects with overweight and obesity, the heterozygote of the c.47 T>C (p.Val16Ala)–*SOD2* (rs4880) polymorphism was associated with an increased risk of urolithiasis occurrence (Crude OR 1.959; 1.022–3.754 95% CI; *p* < 0.05), whereas the C/C genotype of the same polymorphism reduced this risk (Crude OR 0.390; 0.169–0.898 95% CI; *p* < 0.05). In the group with normal body weight, we did not observe these associations.

### SNPs of the *IL6*, *IL-8*, *SOD2*, *NOS2* genes and the age of the first urolithiasis attacks, and the number of urolithiasis attacks

We analysed the link between the age distribution of the first depressive episode and the genotypes of all examined SNPs. We did not find any significant differences in the distribution of genotypes and the age of the first urolithiasis attacks (S1 Fig).

Moreover, we also study the impact of the SNP of genes encoding interleukins and enzymes involved in oxidative and nitrative stress on the number of urolithiasis attacks. Of all the patients enrolled in the study, we chose those with two attacks and more. Our analysis showed no impact of all studied SNPs on the number of urolithiasis attacks (S2 Fig).

### *IL-6*, *IL-8*, *SOD2*, *NOS2* mRNA level analysis

**Fig 3:**
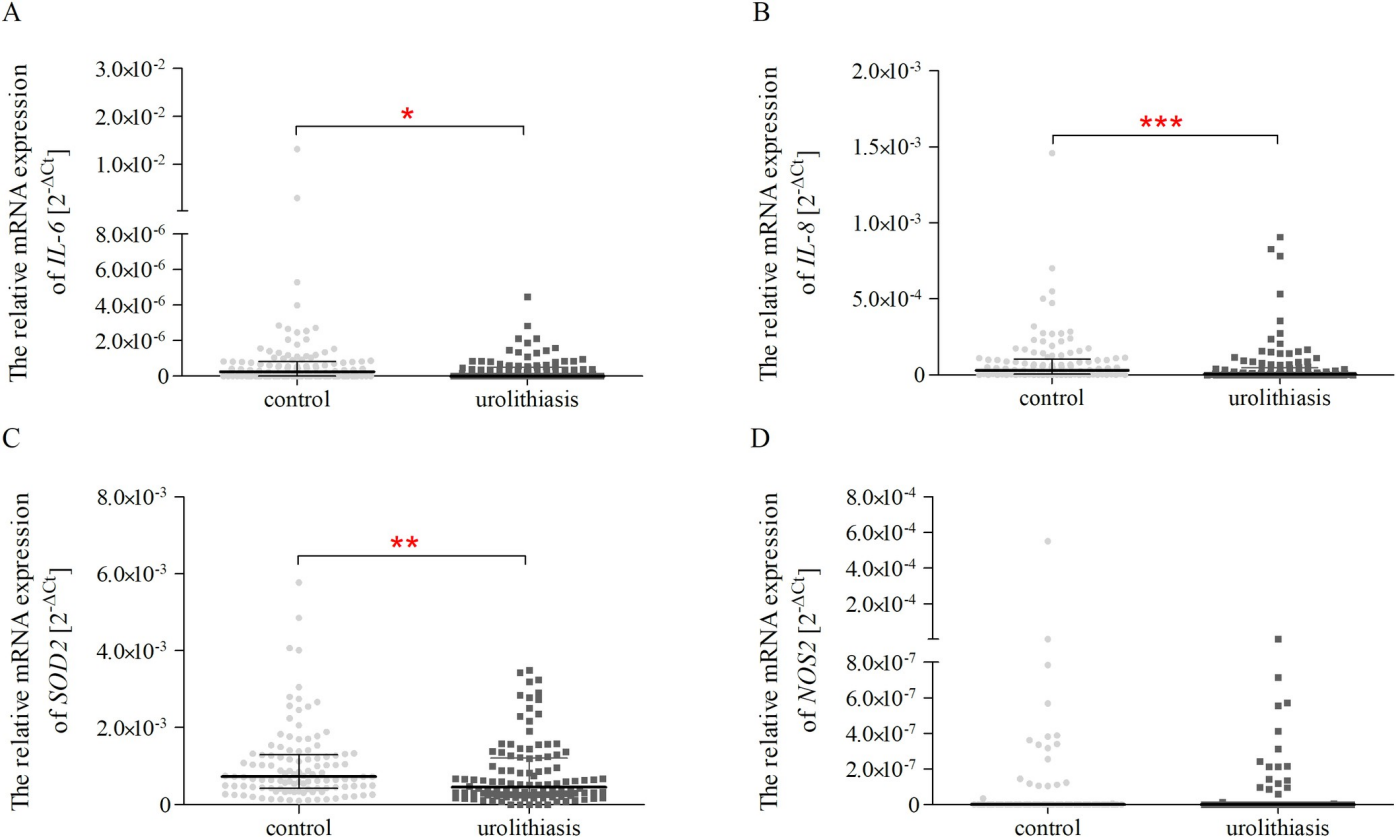
Basal mRNA expression of *IL-6* (A), *IL-8* (B), *SOD2* (C), and *NOS2* (D) genes in PBMCs of controls (N _control_ = 114) and patients with urolithiasis (N _patients with urolithiasis_ = 112). Relative gene expression levels were calculated by the 2^−ΔCt^ (ΔC_t_ = Ct target gene−C_t 18S_) method. The data are plotted as individual values and the median with an interquartile range is indicated by the horizontal bars; *–*p* < 0.05; **–*p* < 0.01; ***–*p* < 0.001.

Analysis of mRNA expression of *IL-6* (*p* < 0.05), *IL-8* (*p* < 0.001), and *SOD2* (*p* < 0.01) showed a significant decrease in mRNA level in patients with urolithiasis compared to the controls (Fig 3). Moreover, there were no differences in *NOS2* mRNA expression levels in subjects with urolithiasis when compared to the healthy volunteers (Fig 3).

### *IL-6*, *IL-8*, *SOD2*, and *NOS2* expression in patients with the first episode of renal colic and patients with recurrent stones

We also performed an additional analysis of the expression level, by extracting patients with the first and subsequent attacks of renal colic. Unfortunately, statistical analysis showed no significant differences between patients with the first attack of renal colic and patients with recurrent disease (S3 Fig).

### *IL-6*, *IL-8*, *SOD2*, *NOS2* expression in the genotype groups

**Fig 4:**
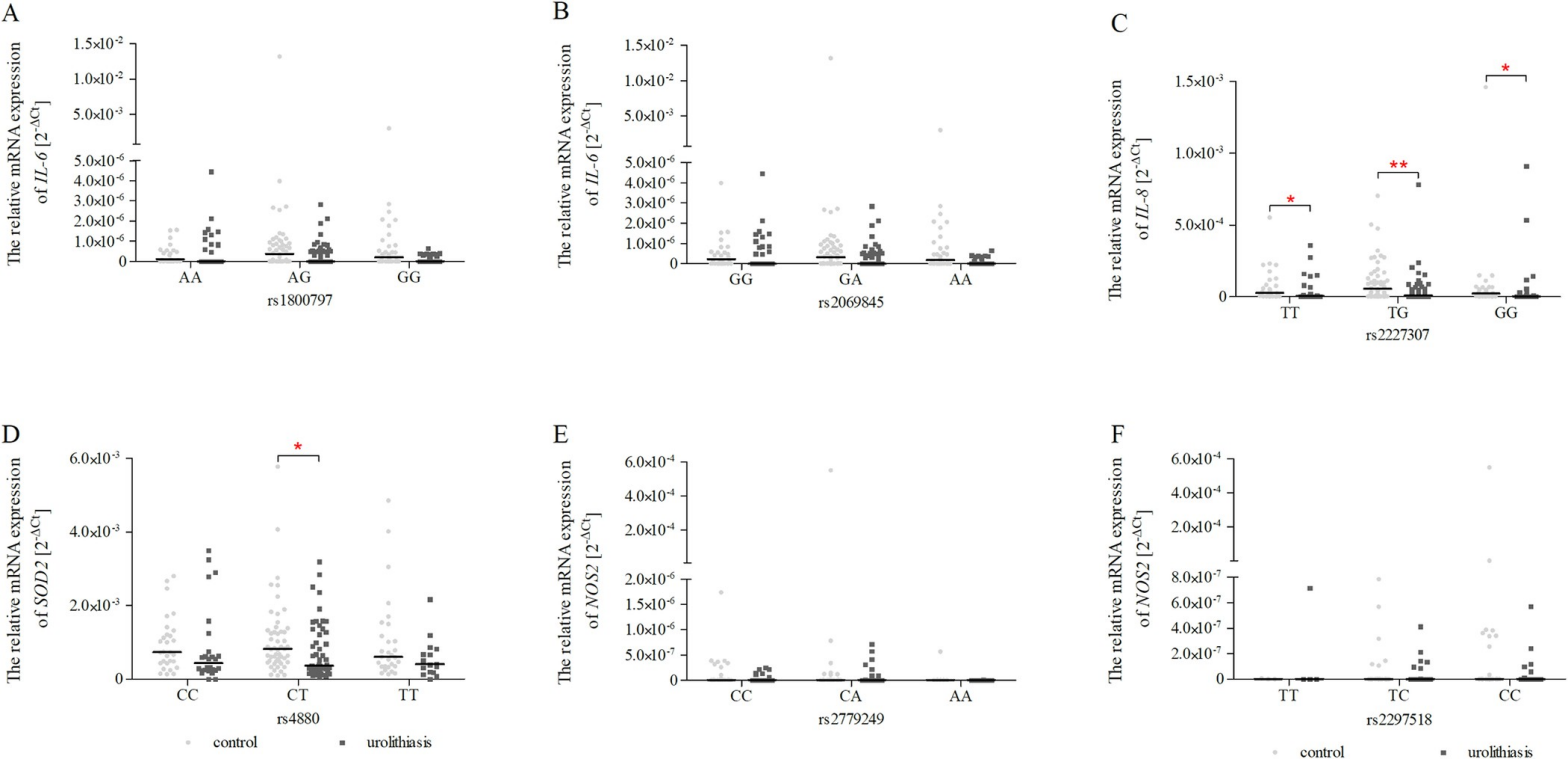
Relative *IL-6*, *IL-8*, *SOD2*, and *NOS2* gene expression in PBMCs in the genotype groups, expressed as 2^−ΔCt^ (ΔC_t_ = C_t target gene_−C_t 18S_) method for each sample. The data are plotted as individual values and the median with an interquartile range is indicated by the horizontal bars; *–*p* < 0.05; **–*p* < 0.01; ***–*p* < 0.001.

Variation in mRNA expression plays a key role in ensuring the phenotypic diversity of the human population. This phenotypic diversity may be the result of the appearance of various polymorphic variants that affect the change of the protein-coding sequence or at the RNA level, modulate the course of the transcription process (activation or inhibition through regulatory sites or the structure of regulatory elements), mRNA processing, mRNA pre-splicing, enhancers exonic splicing (ESE), exon skipping and regulatory RNA [37]. Therefore, the presented study also examines the influence of the genotypes of the tested polymorphisms on the level of mRNA expression. Unfortunately, this analysis did not reveal an effect of genotypes for each studied SNPs on mRNA expression of *IL-6*, *IL-8*, *SOD2*, and *NOS2* (S4 Fig). Moreover, we have performed an additional analysis of *IL-6*, *IL-8*, *SOD2*, and *NOS2* expression in the genotype groups of controls and patients with urolithiasis. We observed that T/T (*p* < 0.05) and G/G homozygotes (*p* < 0.05), as well as heterozygotes (*p* < 0.05) of c.+396 T>G–*IL-8* (rs2227307) SNP, were characterised by reduced *IL-8* expression in the patients with urolithiasis compared with controls (Fig 4C). Moreover, in the case of the c.47 T>C (p.Val16Ala)–*SOD2* (rs4880) polymorphism, we found a decreased *SOD2* mRNA expression in heterozygote patients with urolithiasis compared with controls (Fig 4D). For the left-over analysed SNPs, no statistically significant differences were observed (Fig 4A, 4B, 4E and 4F).

### Effect of gender/BMI and urolithiasis on the mRNA expression of *IL-6*, *IL-8*, *SOD2*, and *NOS2*

**Fig 5:**
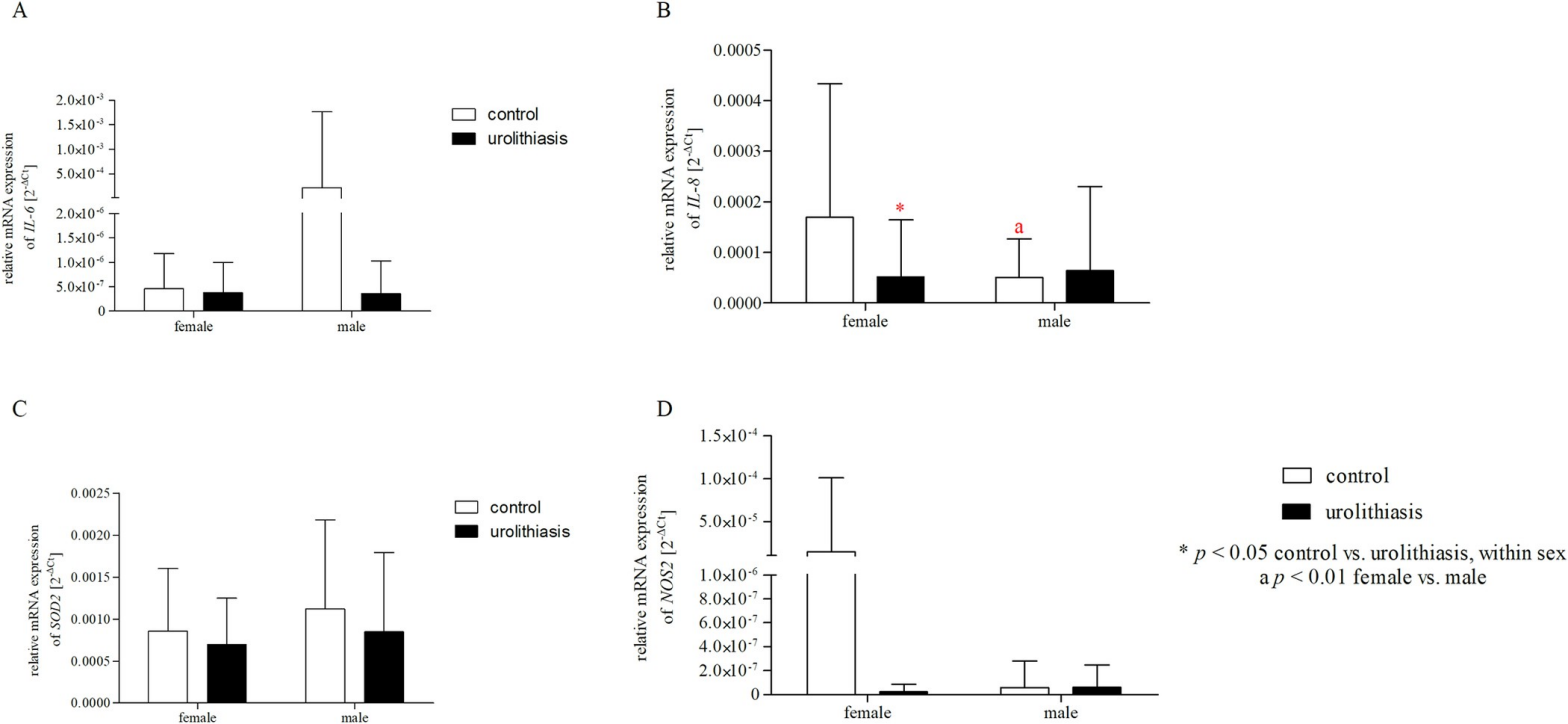
Two-way ANOVA with Bonferroni post-hoc test shows significant effects of gender and urolithiasis on the mRNA expression of *IL-6* (A), *IL-8* (B), *SOD2* (C), and *NOS2* (D). Gene expression in PBMCs has been expressed as 2^−ΔCt^ (ΔC_t_ = Ct _target gene_−Ct _18S_) method. The data are presented as mean ± SD.

**Fig 6:**
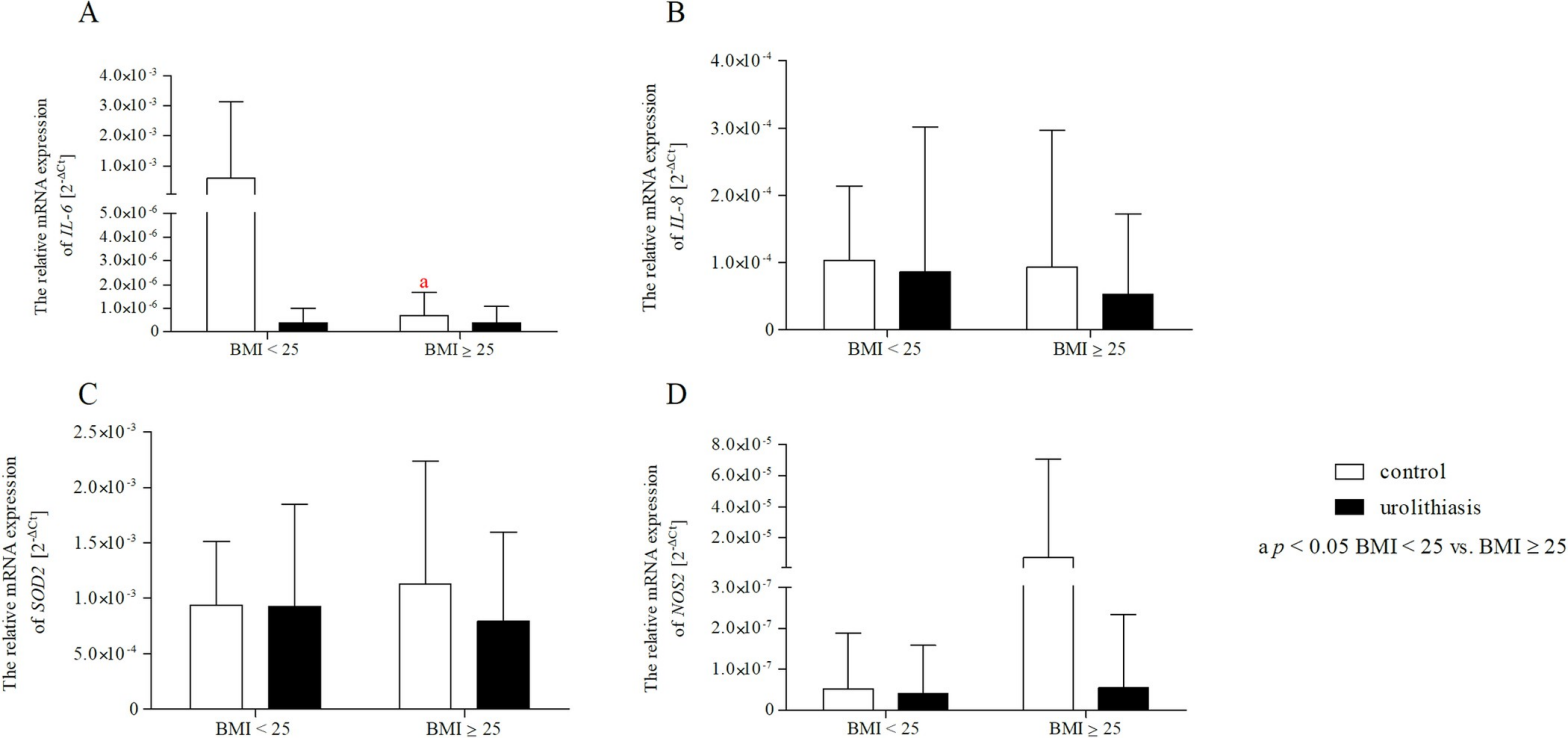
Two-way ANOVA with Bonferroni post-hoc test shows significant effects of BMI and urolithiasis on the mRNA expression of *IL-6* (A), *IL-8* (B), *SOD2* (C), and *NOS2* (D). Gene expression in PBMCs has been expressed as 2^−ΔCt^ (ΔC_t_ = Ct _target gene_−Ct _18S_) method. The data are presented as mean ± SD.

Our additional analysis (Table 11) showed a significant effect of gender and BMI on the differences in the expression level of IL-8 (p < 0.05) and IL-6 (p < 0.05), respectively, between the control group and urolithiasis. In addition, we detected significant effects of the gender × group interaction for IL-8 expression (p < 0.01) and the BMI × group interaction for IL-6 expression (p < 0.05). Interestingly, further analysis using the Bonferroni post-hoc test (Fig 5) showed that the expression level of IL-8 mRNA was significantly lower in the subgroup of men than in women in the control group (p < 0.01). In turn, patients with urolithiasis showed a reduced expression of IL-8 mRNA compared to healthy volunteers, but only in a subgroup of women (p < 0.05). Moreover, in the case of BMI analysis, a two-way ANOVA with the post-hoc Bonferroni test (Fig 6) confirmed that subjects with a BMI above the norm showed a lower level of IL-6 expression than subjects with a normal BMI in a subgroup of healthy volunteers (p < 0 .05).

**Table 11:** Results of two-way ANOVA analyses on mRNA expression of *IL-6*, *IL-8*, *SOD2* and *NOS2*.

Factor	Genes	Study groups		Gender/BMI		Interaction	
		F	*p*	F	*p*	F	*p* *
Gender	*IL-6*	0.722	0.396	0.720	0.397	0.721	0.397
	***IL-8***	**5.283**	**0.022**	**5.481**	**0.020**	**8.375**	**0.004**
	*SOD2*	2.831	0.094	2.564	0.111	0.193	0.661
	*NOS2*	1.881	0.172	1.862	0.174	1.882	0.172
BMI	***IL-6***	**4.434**	**0.036**	**4.424**	**0.037**	**4.425**	**0.037**
	*IL-8*	1.204	0.274	0.688	0.408	1.196	0.658
	*SOD2*	1.474	0.226	0.043	0.837	1.244	0.266
	*NOS2*	0.388	0.534	0.388	0.534	0.385	0.535

*p < 0.05 are in bold

## Discussion

Urolithiasis is one of the most common urological diseases with a worldwide incidence of 1.7–14.8% depending on the geographic and socioeconomic characteristics of different populations, and over $ 2 billion is spent on treatment each year [4, 38]. Unfortunately, the mechanism of urolithiasis development remains unclear. However, a growing body of evidence suggests that urolithiasis is a multifactorial disease, most probably caused by a complex two-way environment-gene interaction. Despite the fact that the main risk factors for the development and recurrence of urolithiasis are overweight, obesity, diabetes, low fluid intake, high salt diet, excessive intake of calcium, animal protein, oxalate, sodium, potassium, magnesium, and sucrose and limited physical activity studies involving twins confirm the importance of genetic factors in the formation of deposits in the urinary tract [20, 39]. Interestingly, kidney stones’ family history has been reported in 4–12% of healthy controls compared with 16–37% of renal colic affected. Furthermore, previous studies showed that urinary stones develop about three times more often in people with a positive family history [19, 40, 41]. Among heritable traits that might contribute to the overall heritability of urinary stone disease should be mentioned urinary composition, dietary risk factors, calcium and vitamin D homeostasis, and metabolic syndrome traits [42]. However, despite numerous reports pointing to possible causes of plaque formation in the urinary tract, the molecular mechanism of urolithiasis development is still unclear, and previous studies have focused only on the primary role of environmental causes of the disease [39]. Nevertheless, there are growing new reports that point out the crucial role of both inflammation as well as nitrative and oxidative stresses in urolithiasis development [43–50].

Previous studies showed that renal deposits can stimulate renal epithelial and immune cells to secrete inflammatory mediators, including monocyte chemoattractant protein-1 (MCP-1), tumour necrosis factor-alpha (TNF-α), IL-6, IL-8, and C-reactive protein (CRP) [51–54]. On the other hand, inflammation can be further exacerbated by the overproduction of ROS and RNS that are generated during the formation of urinary deposits. The primary source of ROS in kidneys is the activity of NADPH oxidase, over-activated by angiotensin II, which is produced by cells activated by renal crystals [23]. Consequently, ROS and RNS activate the transcription factors (such as NF-κB) of isoprostanes and prostaglandins genes, leading to the modulation of inflammation. ROS-induced NF-κB can also regulate the expression of genes encoding adhesion molecules, *COX-2* and pro-inflammatory cytokines, *TNF-α*, *IL-6*, and *CRP*. Then, in a vicious circle mechanism, these proinflammation factors may enhance NADPH oxidase activation and may additionally stimulate the production of ROS, leading to impaired endothelial function [55, 56].

As above mentioned, a growing body of evidence suggests that genetic factors can be one of the factors, that play a key role in urolithiasis development. Many chromosomal regions/gene loci associated with renal deposit formulation risk have been identified in genome-wide association studies (GWAS). To date, five genome-wide association studies on nephrolithiasis have been published identifying 15 disease-related loci; however, no trans-ethnic research has been undertaken [57]. The GWAS studies published in 2007–2017 disclosed seven loci associated with propensity to the form of urinary stones, such as 5q35,3 (rs11746443; rs12654812 in *SLC34A1*); 3q21.1 (rs7627468 in *CASR*); 13q14.1 (rs4142110 in *DGKH*); 21q22.13 (rs219780, rs199565725 in *CLDN14*); 7q14.3 (rs1000597, rs12669187 in *AQP1*); 7q34 (p.L530A in *TRPV5*) and 1p36.12 (rs1256328 in *ALPL*) [58]. Moreover, the latest kidney stone-related GWAS reported in an Eastern European population showed three loci as candidates with a highly significant association with nephrolithiasis: (i) rs1118528 in the gene, encoding a mitochondrial ATP-Mg/phosphate carrier protein; (ii) rs4437026 causing TOX2 upregulation in several tumour types and l tumour progression; (iii) and rs10917682 localised in regulator of G protein signalling 5, playing crucial roles in the development of renal cell carcinoma [57]. On the other hand, in the Japanese population GWAS study identified additional crucial loci linked with urolithiasis, including rs6667242, rs11746443, rs3798519, and rs74956940 on 1p36.12, 5q35.3, 6p12.3, and 19p13.12, respectively, for rs1697420, rs10866705, rs62405419, and rs2241358. These SNPs included *GCKR-C2orf16-ZNF512-CCDC121-GPN1-SUPT7L-SLC4A1AP-MRPL33-RBKS* (2p23.2–3), *SAYSD1-KCNK5* (6p21.2), *TFAP2D-TFAP2B* (6p12.3), *EPB41L2* (6q23.2), *PDILT* (16p12.3), *FTO* (16q12.2), *BCAS3-TBX2-C17orf82* (17q23.2), *PKN1-PTGER1-GIPC1* (19p13.12), and *BCAS1* (20q13.2). rs219780 on *CLDN14* [53].

However, despite abundant evidence suggesting a significant role of inflammation as well as oxidative and nitrative stresses in urolithiasis development, the literature review shows that only very few data available points to the association of polymorphisms located in genes involved in these pathways and the modulation of urolithiasis occurrence risk [23, 51–56]. Thus, the presented study was undertaken to the identification of the potential association of six SNPs in inflammation as well as nitrative and oxidative stress-related genes: *IL-6* (-597 A>G, rs1800797; c.3331 G>A, rs2069845), *IL-8* (c.+396 T>G, rs2227307), *SOD2* (c.47 C>T; rs4880) and *NOS2* (c.1823 C>T, rs2297518, g.-1026 C>A, rs2779249), and the urolithiasis occurrence. Moreover, we also analysed the mRNA expression of all studied genes in the control group and patients with urolithiasis. The identification of genes associated with urolithiasis is important to understand better the development of this disease and develop new diagnostic strategies.

According to the data presented above, the deposits forming in the urinary tract are accompanied by the development of inflammation [51–54]. Thus, our presented study contained the evaluation of *IL-6* and *IL-8* polymorphism impact on urolithiasis occurrence. Both IL-6 and IL-8 belong to the group of pro-inflammatory cytokines [55]. In the innate immune response, IL-6 is synthesised by myeloid cells, including macrophages and dendritic cells, at the site of infection or tissue damage after pathogens are recognized by Toll-like receptors (TLRs). Moreover, in the adaptive immune response, IL-6 is necessary for B cell differentiation into immunoglobulin-secreting cells [50, 55, 59, 60]. In turn, IL-8 is a chemokine produced by macrophages, airway smooth muscle cells, epithelial and endothelial cells. It induces chemotaxis of neutrophils and other granulocytes toward the site of infection, where it additionally stimulates phagocytosis [55]. Interestingly, we were the first who analysed the impact of *IL-6* and *IL-8* SNPs on the risk of urolithiasis occurrence. According to the Variation Viewer of the National Center for Biotechnology Information (NCBI), 2603 SNPs present in the Single Nucleotide Polymorphism Database (dbSNP) and 115 various mutations (including copy number variation, deletion, insertion, short tandem repeat variation, inversion, mobile element insertion and tandem duplication) in *IL-6* have been registered, whereas *IL-8* has 1509 SNPs and 133 other mutations (including copy number variation, deletion, insertion, indel, inversion, tandem duplication) [61]. However, previous reports have focused on the frequency of polymorphisms located in *IL-1RN*, *IL-1β*, and *IL-18* genes in patients with urolithiasis. Thus, the first analysed polymorphism in our study is (-597 A>G–*IL-6* (rs1800797) localised in the promoter region on chromosome 7. Bennermo et al. (2004) found that individuals with the G allele of this SNP were characterised by an increased inflammatory response [30]. The second studied SNP localised in the intron of the *IL-6* gene is c.3331 G>A (rs2069845). As in the case of the previous *IL-6* polymorphism, also in this SNPs G-carriers exhibited increased transcription of the gene relating to the elevated secrete IL-6 in serum [30]. The c.+396 T>G (rs2227307) polymorphism of *IL-8* is localised in the intron on chromosome 4. Interestingly, SNPs in the gene intron may modulate the mRNA/protein splicing process, leading to protein isoform formation [51, 62]. On the other hand, our analyses did not confirm the influence of the tested SNPs on the expression level of *IL-6* and *IL-8*. However, in the case of G-carriers of c.3331 G>A (rs2069845) SNP, we observed a non-significant increase of *IL-6* expression. Unfortunately, our study did not show any association between -597 A>G SNP in the *IL-6*, c.3331 G>A SNP in the *IL-6*, c.+396 T>G SNP in the *IL-8* and the urolithiasis occurrence. However, SF analysis confirmed the existence of antagonistic interaction between c.+396 T>G–*IL-8* (rs2227307) and c.3331 G>A–*IL-6* (rs2069845). Moreover, in our study, we showed that patients with urolithiasis were characterised by reduced *IL-6* and *IL-8* expression in PBMCs. On the other hand, the earlier study confirmed that the kidney tissue of patients with urolithiasis exhibited a higher mRNA expression of *IL-6* than healthy volunteers [63]. In addition, another study affirmed that urolithiasis patients were characterised by elevated IL-6 and IL-8 levels in the urine compared with controls [55]. Most likely, it is the result of increased production of IL-6 and IL-8 at the site of damage, i.e. in the urinary tract. Accordingly, increased release of IL-6 and IL-8 in the urinary system confirms increased levels of these cytokines in the urine [55].

As mentioned above, inflammation may develop as a result of the formation of a deposit, or it may be a consequence of the intensification of oxidative stress processes observed in the course of urolithiasis [64]. Therefore, in our study we also analysed polymorphism located in *SOD2*, encoding superoxide dismutase 2. SOD2 is mitochondrial manganese superoxide dismutase (MnSOD), which is a crucial cell element of the antioxidant defence. An increased SOD2 activity causes an elevated level of H_2_O_2_, which is then neutralised by catalase. Thus, polymorphic variants may play a crucial role in the modulation of SOD2 activity, leading to many disease development, including disorders of the urinary tract [65, 66]. Interestingly, previous data of Variation Viewer of the NCBI confirmed 46490 registered mutations localised within or near the vicinity of *SOD2*. These include 45778 SNPs present in the Single Nucleotide Polymorphism Database (dbSNP) and 712 various mutations, i.e. copy number variations, deletion, insertions, short tandem repeat variations, indel, inversions, mobile element insertions, and tandem duplications, listed in the Database of Genomic Structural Variation (dbVar) [61]. According to the literature data, the c.47 C>T (Ala9Val) polymorphism is the most frequently studied. Interestingly, previous results suggest that the T allele of the c.47 C>T (Ala9Val) polymorphism decreased the expression and production of an unstable mRNA, which affects the reduction of its antioxidant potential in mitochondria [67, 68]. However, in opposition to the discoveries of Tugcu’s team (2007), we found that the T/T genotype was associated with a reduced risk of kidney stones, while heterozygotes increased this risk [45]. Moreover, additional analysis, including smoking cigarettes and BMI showed that the heterozygote showed an elevated risk of urolithiasis in only smokers and BMI ≥ 25 groups, whereas the T/T homozygote reduced this risk in only smokers and BMI ≥ 25 groups. We also detected that *SOD2* expression in PBMCs was lower in patients with urolithiasis than in controls. Similarly, a paediatric population study demonstrated that kidney stone formation was associated with decreased antioxidant enzyme activity, including superoxide dismutase, glutathione peroxidase, and glutathione-S-transferase. The reduced enzyme activity worsened the urolithiasis course due to the organism’s inability to counter the damaging effects of ROS [46, 69]. Moreover, our additional analysis of expression in PBMCs in the genotype groups confirmed previous discoveries that T/T groups were characterised by the lowest *SOD2* expression, however, our result was not statistically significant [67, 68]. These differences in the nature of the interaction of *SOD2* genotypes while maintaining reduced expression for T-carriers in the presented studies may result from alternative mechanisms of gene expression regulation, e.g. epigenetic modifications, including DNA methylation and chromatin histone modifications which effectively silence gene expression [69].

Oxidative stress is inevitably associated with nitrative stress, thus, in the presented manuscript we also analysed polymorphisms located in *NOS2*. NOS2 is also known as Inos and belongs to the NO synthase family. *NOS2* expressed is stimulation by pro-inflammatory cytokines and the same enzyme converts the L-arginine to NO. The overproduction of NO leads to an imbalance and the development of nitrative stress [45]. The number of known mutations located in *NOS2* in Variation Viewer of the NCBI database, which is 19359, seems impressive. These include 19541 single-nucleotide variants present in the NCBI dbSNP database and 182 various mutations, including copy number variations, inversions, short tandem repeat variations, insertions, and mobile element insertions, listed in the NCBI dbVar database [61]. Thus, in our study, we analysed two *NOS2* polymorphisms, including c.1823 C > T (rs2297518) and g.-1026 C>A (rs2779249). Previous studies confirmed that the substitution in the case of both SNPs is associated with the increase of NOS2 activity, leading to the overproduction of NO [33]. Unfortunately, we did not show any association between both studied polymorphisms and the urolithiasis occurrence. Similarly, in the case of analysis of the impact of the tested polymorphic variants on the level of NOS2 expression, we did not demonstrate a significant effect, however, the appearance of these SNPs non-statistically increased the level of NOS2 expression, which suggests that perhaps after increasing the number of tested groups, this relationship will be confirmed statistically. An additional SNP analysis, including gender, showed that the C/A genotype of the g.-1026 C>A (rs2779249) SNP reduced the urolithiasis risk in only the women population. In turn, in the case of NOS2 expression in PBMCs, we also found no statistical decrease in patients with urolithiasis as compared to controls. On the other hand, Huang et al. (2006) found that nephrolithiasis may be associated with an increase in *Inos* expression in the renal medulla of the ethylene glycol (EG)-treated rat, which is known as the established model for deposit formation in kidneys [24]. These differences may result from the different material that was analysed in both studies. It may also suggest that the changes due to deposit formation in kidneys (renal medulla) do not correspond to the peripheral change observed in PBMCs.

Interestingly, our results indicate for the first time that urolithiasis risk may be modulated not only by a single locus with genetic main effects but also by epistatic (gene-gene) interactions in studied genes. Obtained results showed an association between two two-gene combinations and the increased risk of urolithiasis: rs1800797–rs4880, rs4880–rs2069845.

As already mentioned, the increasing number of new cases of urolithiasis and its recurrent nature related to lifestyle changes have recently become a serious problem in developed countries. Moreover, preventive measures related to the use of an appropriate diet are usually introduced too late, only after the first episode of renal colic [1–4]. Therefore, the ability to predict urolithiasis in people at risk using molecular markers can bring enormous benefits to patients, as well as reduce the sociological and financial burden of the disease, and may also help avoid the development of severe complications of urolithiasis, including bladder cancer. However, the heterogeneous nature of the disease makes it very difficult to create a universal panel of such markers. Moreover, recently, in addition to environmental, sociodemographic and genetic factors, the key role of epigenetics in the pathogenesis of urolithiasis has been emphasized [70, 71]. Our results show that the tested SNPs located in genes involved in the regulation of oxidative and nitrative stress and inflammation, as well as impaired mRNA expression of these genes, are associated with the occurrence of urolithiasis. Therefore, this work is part of the current research trend, which aims to develop an effective panel of prognostic markers. Nevertheless, our research should be expanded with further analyses, including epigenetic modifications, preferably with the participation of different ethnic groups, and then confirmed by meta-analysis, which may contribute to the creation of multigene risk scales in the future.

We have as first shown that studied polymorphisms, localised in the genes associated with inflammation as well as oxidative and nitrative stresses may have an impact on the risk of urolithiasis development. However, it ought to be borne in mind that each study, including ours, has limitations. First, the presented study is preliminary, limited to a single population, and relatively small sample size, which in turn gives the possibility that the results may not be duplicated in other populations. The next limitation is the ethnic origin of the study participants. Therefore, one should remember that our results cannot be freely extrapolated to other ethnic groups. The presented limitations are a consequence of the specificity of the analysed material and its limited availability. Nevertheless, our study needs research on more patients in the future.

## Conclusion

To conclude, our presented study showed a significant association between genes involved in inflammation as well as oxidative and nitrative stresses and the occurrence of urolithiasis. These results might help to identify specific molecular markers of urolithiasis and develop new diagnostic strategies.

In conclusion, obtained data suggest that reduced *IL-6*, *IL-8*, and *SOD2* mRNA expression as well as the c.47 C>T (rs4880) polymorphism of *SOD2* are associated with urolithiasis. Thus, inflammation as well as oxidative and nitrative stress genes can play a crucial role in the pathogenesis of urolithiasis.

## Supporting information

S1 Fig Distribution of single nucleotide polymorphisms of genes encoding IL-6 (A, B), IL-8 (C), SOD2 (D), NOS2 (E, F) and the age of the first renal colic attack. The data are plotted as individual values and the median with an interquartile range is indicated by the horizontal bars.
(TIF)

S2 Fig Distribution of single nucleotide polymorphisms of genes encoding IL-6 (A, B), IL-8 (C), SOD2 (D), NOS2 (E, F) and the number of renal colic attacks. The data are plotted as individual values and the median with an interquartile range is indicated by the horizontal bars.
(TIF)

S3 Fig Basal mRNA expression of IL-6 (A), IL-8 (B), SOD2 (C), and NOS2 (D) genes in PBMCs of patients with the first attack of renal colic (1 episode) and patients with recurrent disease (> 1 episode). Relative gene expression levels were calculated by the 2−ΔCt method (ΔCt = Ct target gene−Ct 18S) method. The data are plotted as individual values and the median with an interquartile range is indicated by the horizontal bars.
(TIF)

S4 Fig Distribution of single nucleotide polymorphisms of genes encoding IL-6 (A, B), IL-8 (C), SOD2 (D), NOS2 (E, F) and mRNA expression level of IL-6, IL-8, SOD2, NOS2 expressed as 2−ΔCt (ΔCt = Ct target gene−Ct 18S) method for each sample. The data are plotted as individual values and the median with an interquartile range is indicated by the horizontal bars.
(TIF)

S1 Table Gene-gene interactions of studied inflammation and oxidative stress-related polymorphisms and urolithiasis risk. (PDF)

S2 Table Distribution of haplotypes of the studied polymorphisms of the *IL-6* or *NOS2* genes and risk of urolithiasis. (PDF)

S3 Table Distribution of genotypes and alleles of the -597 A>G–*IL-6* (rs1800797), c.3331 G>A–*IL-6* (rs2069845), c.+396 T>G–*IL-8* (rs2227307), c. 47 C>T–*SOD2* (rs4880), c.1823 C>T (p. Ser608Leu)–*NOS2* (rs2297518) and ORs with 95% CIs in men and women with urolithiasis. (PDF)

S4 Table Distribution of genotypes and alleles of the -597 A>G–*IL-6* (rs1800797), c.3331 G>A–*IL-6* (rs2069845), c.+396 T>G–*IL-8* (rs2227307), c.1823 C>T (p. Ser608Leu)–*NOS2* (rs2297518), g.-1026 C>A–*NOS2* (rs2779249) and ORs with 95% CIs in non-smokers and smokers. (PDF)

S5 Table Distribution of genotypes and alleles of the -597 A>G–*IL-6* (rs1800797), c.3331 G>A–*IL-6* (rs2069845), c.+396 T>G–*IL-8* (rs2227307), c.1823 C>T (p. Ser608Leu)–*NOS2* (rs2297518), g.-1026 C>A–*NOS2* (rs2779249) and ORs with 95% CIs in subjects with normal body weight or subjects with overweight and obesity. (PDF)
