# Supplementary figures and images for: Polymorphic variations and mRNA expression of the genes encoding interleukins as well as enzymes of oxidative and nitrative stresses as a potential risk of nephrolithiasis development
Source: PLoS One. 2023 Oct 25;18(10):e0293280. doi: 10.1371/journal.pone.0293280 (PMC10599546; doi:10.1371/journal.pone.0293280)

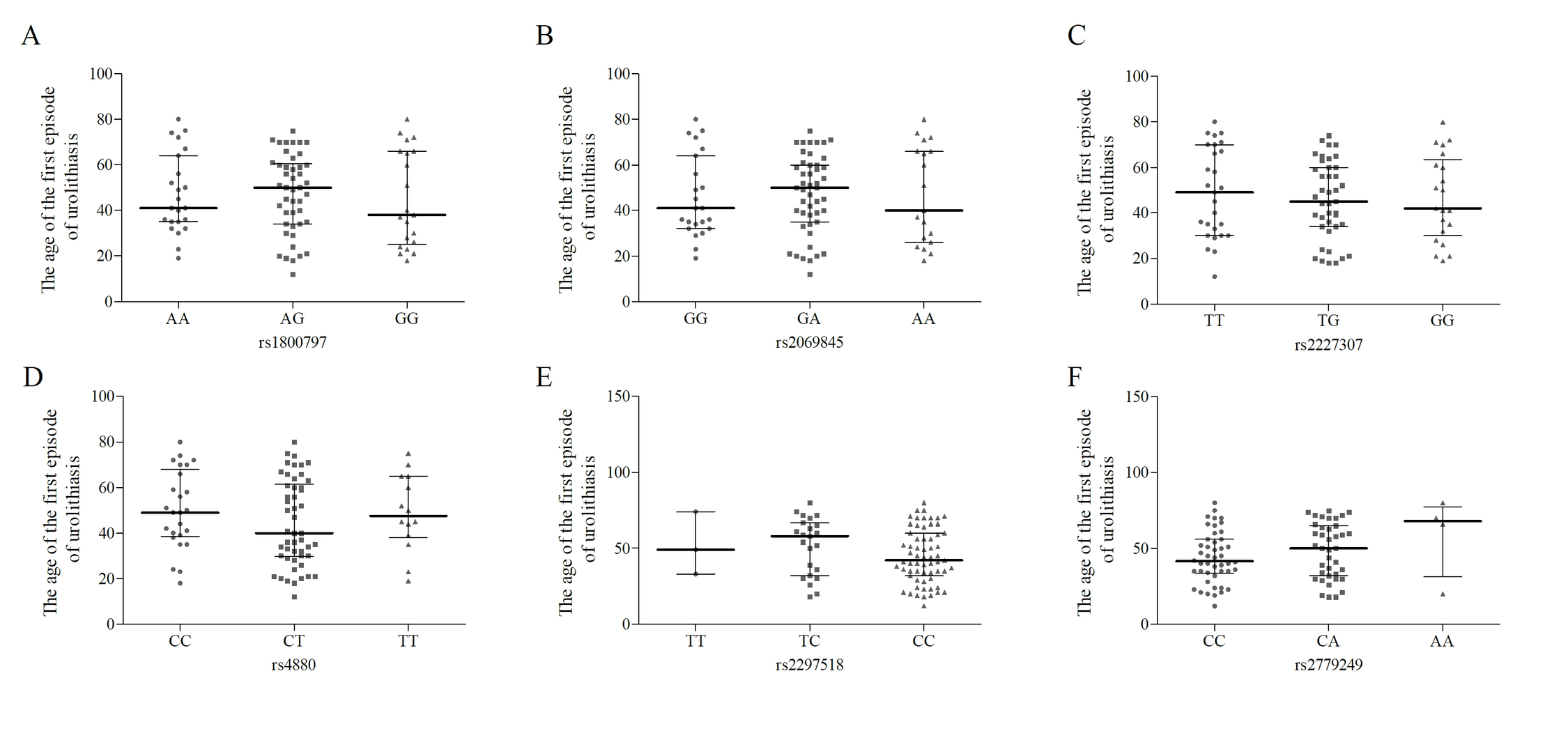

Supplement: S1 Fig — Distribution of single nucleotide polymorphisms of genes encoding IL-6 (A, B), IL-8 (C), SOD2 (D), NOS2 (E, F) and the age of the first renal colic attack. The data are plotted as individual values and the median with an interquartile range is indicated by the horizontal bars. (TIF) [file pone.0293280.s001.tif]

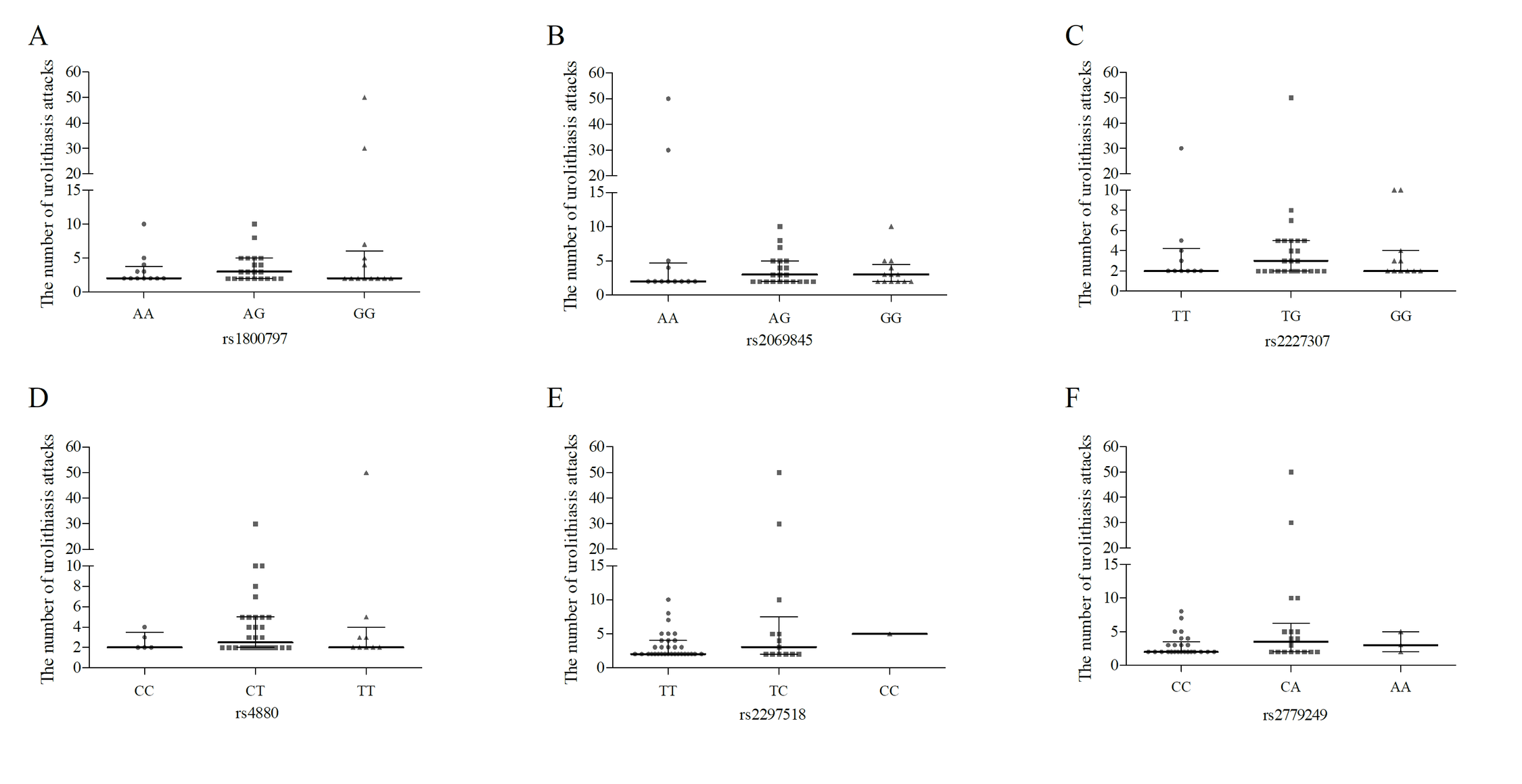

Supplement: S2 Fig — Distribution of single nucleotide polymorphisms of genes encoding IL-6 (A, B), IL-8 (C), SOD2 (D), NOS2 (E, F) and the number of renal colic attacks. The data are plotted as individual values and the median with an interquartile range is indicated by the horizontal bars. (TIF) [file pone.0293280.s002.tif]

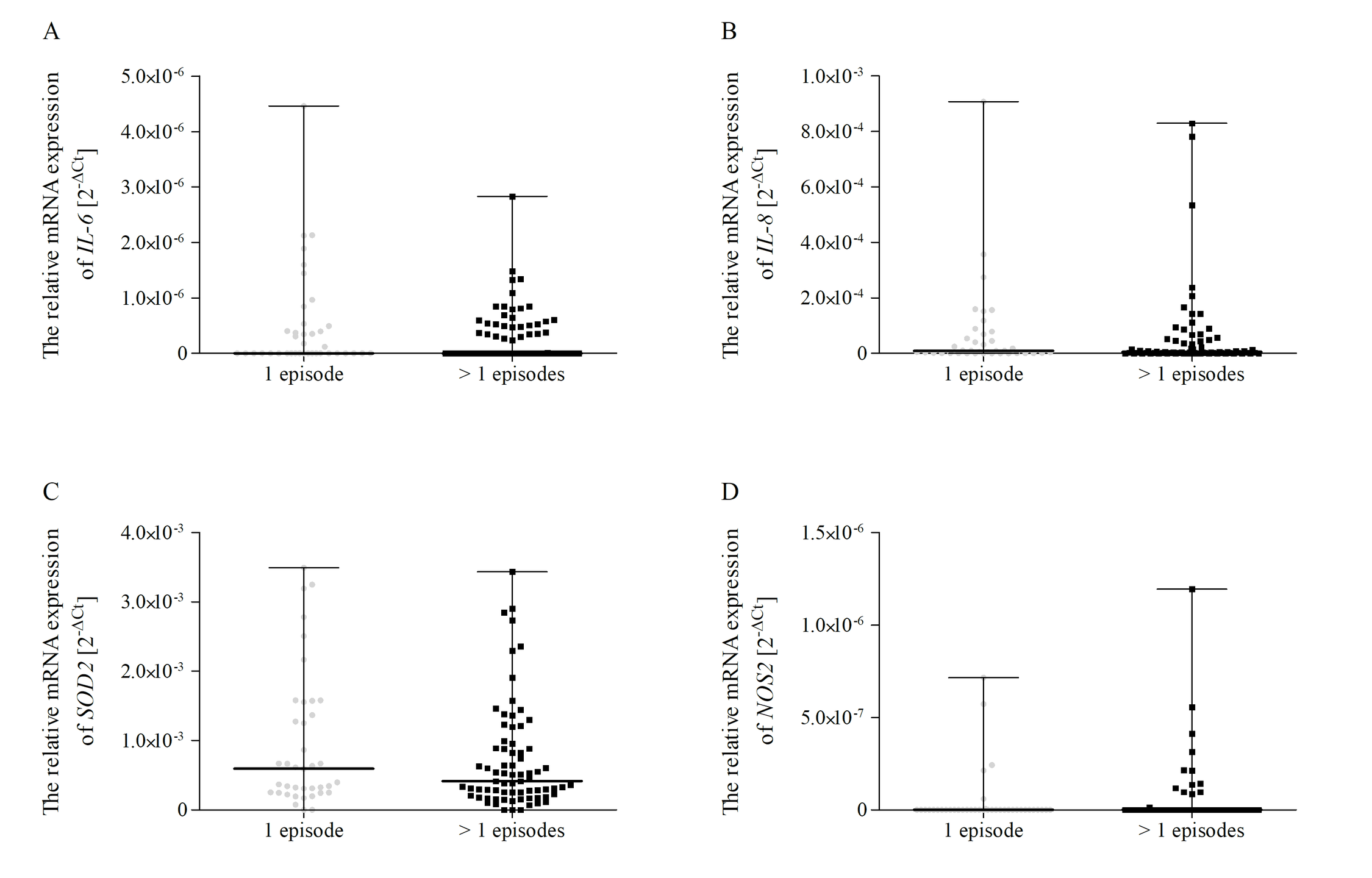

Supplement: S3 Fig — Basal mRNA expression of IL-6 (A), IL-8 (B), SOD2 (C), and NOS2 (D) genes in PBMCs of patients with the first attack of renal colic (1 episode) and patients with recurrent disease (> 1 episode). Relative gene expression levels were calculated by the 2−ΔCt method (ΔCt = Ct target gene−Ct 18S) method. The data are plotted as individual values and the median with an interquartile range is indicated by the horizontal bars. (TIF) [file pone.0293280.s003.tif]

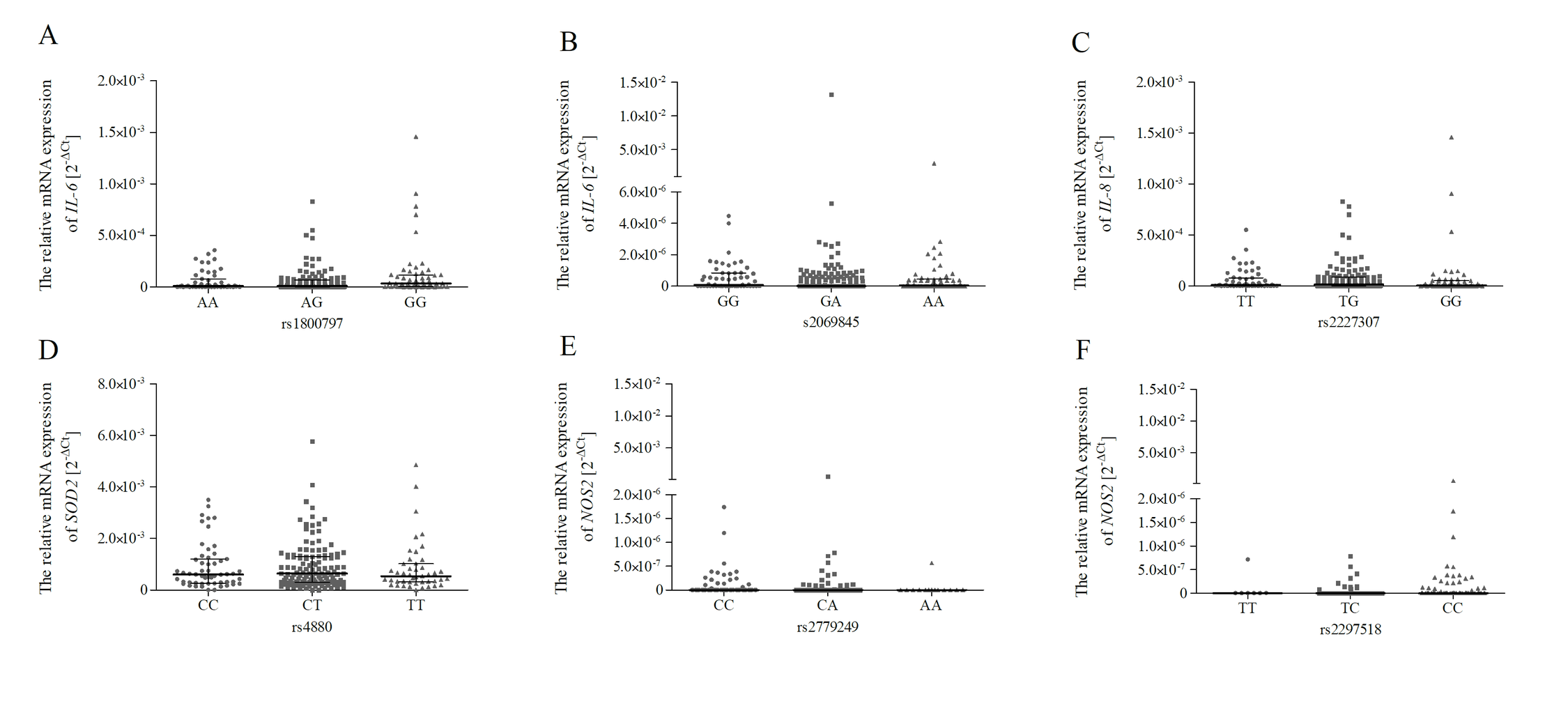

Supplement: S4 Fig — Distribution of single nucleotide polymorphisms of genes encoding IL-6 (A, B), IL-8 (C), SOD2 (D), NOS2 (E, F) and mRNA expression level of IL-6, IL-8, SOD2, NOS2 expressed as 2−ΔCt (ΔCt = Ct target gene−Ct 18S) method for each sample. The data are plotted as individual values and the median with an interquartile range is indicated by the horizontal bars. (TIF) [file pone.0293280.s004.tif]
