# Supplementary material for: Polymorphic variations and mRNA expression of the genes encoding interleukins as well as enzymes of oxidative and nitrative stresses as a potential risk of nephrolithiasis development
Source: PLoS One. 2023 Oct 25;18(10):e0293280. doi: 10.1371/journal.pone.0293280 (PMC10599546; doi:10.1371/journal.pone.0293280)
Supplement: S1 Table — (PDF) [file pone.0293280.s005.pdf]

S1. Table. Gene–gene interactions of studied inflammation and oxidative stress-related polymorphisms and urolithiasis risk.

Combined genotypes	Control (n = 114)		Urotheriasis (n = 112)		Crude OR (95% CI)*	*p*	Adjusted OR (95% CI)*	*p*
	Number	Frequency	Number	Frequency				
**-597 A>G (rs1800797) – *IL-6* and c.1823 C>T (p. Ser608Leu) – *NOS2* (rs2297518)**								
A/A-T/T	1	0.009	1	0.009	1.018 (0.063-16.478)	0.900	0.987 (0.061-16.070)	0.992
A/A-T/C	6	0.063	7	0.063	1.200 (0.390-3.689)	0.750	1.217 (0.395-3.752)	0.773
A/A-C/C	16	0.179	20	0.179	1.332 (0.651-2.725)	0.433	1.325 (0.647-2.714)	0.442
A/G-T/T	1	0.009	1	0.009	1.018 (0.063-16.478)	0.900	0.987 (0.061-16.070)	0.992
A/G-T/C	19	0.167	15	0.134	0.773 (0.372-1.610)	0.492	0.781 (0.374-1.632)	0.510
A/G-C/C	38	0.333	41	0.366	1.155 (0.668-1.996)	0.606	1.147 (0.662-1.987)	0.624
G/G-T/T	1	0.009	2	0.018	2.055 (0.184-22.985)	0.559	2.056 (0.184-23.008)	0.559
G/G-T/C	10	0.088	8	0.071	0.800 (0.304-2.107)	0.652	0.804 (0.305-2.121)	0.660
G/G-C/C	22	0.193	17	0.152	0.748 (0.374-1.499)	0.413	0.750 (0.374-1.504)	0.418
**-597 A>G (rs1800797) – *IL-6* and g.-1026 C>A – *NOS2* (rs2779249)**								
A/A-C/C	12	0.105	17	0.152	1.521 (0.690-3.352)	0.298	1.518 (0.689-3.346)	0.301
A/A-C/A	10	0.088	11	0.098	1.133 (0.461-2.783)	0.786	1.133 (0.461-2.785)	0.786
A/A-A/A	1	0.009	0	0.000	0.000 (0.000-+inf)	0.991	0.000 (0.000-+inf.)	0.991
A/G-C/C	30	0.263	26	0.232	0.847 (0.462-1.550)	0.589	0.831 (0.451-1.532)	0.553
A/G-C/A	26	0.228	26	0.232	1.023 (0.551-1.901)	0.942	1.035 (0.555-1.930)	0.914
A/G-A/A	2	0.018	5	0.045	2.617 (0.497-13.778)	0.256	2.646 (0.502-13.956)	0.251
G/G-C/C	15	0.132	14	0.125	0.943 (0.432-2.057)	0.882	0.951 (0.435-2.079)	0.900
G/G-C/A	15	0.132	10	0.089	0.647 (0.278-1.509)	0.314	0.646 (0.277-1.507)	0.312
G/G-A/A	3	0.026	3	0.027	1.018 (0.201-5.156)	0.982	1.019 (0.201-5.159)	0.982
**-597 A>G – *IL-6* (rs1800797) and c.+396 T>G – *IL-8* (rs2227307)**								
A/A-T/T	4	0.035	10	0.089	2.696 (0.820-8.866)	0.102	2.686 (0.816-8.836)	0.104
A/A-T/G	11	0.096	13	0.116	1.230 (0.526-2.874)	0.633	1.225 (0.524-2.864)	0.640
A/A-G/G	8	0.070	5	0.045	0.619 (0.196-1.954)	0.414	0.622 (0.197-1.964)	0.418
A/G-T/T	12	0.105	13	0.116	1.116 (0.486-2.565)	0.796	1.106 (0.480-2.547)	0.813
A/G-T/G	33	0.289	32	0.286	0.982 (0.552-1.747)	0.950	0.989 (0.555-1.762)	0.969
A/G-G/G	13	0.114	12	0.107	0.932 (0.406-2.142)	0.869	0.923 (0.401-2.125)	0.851
G/G-T/T	11	0.096	5	0.045	0.438 (0.147-1.303)	0.138	0.441 (0.148-1.315)	0.142
G/G-T/G	17	0.149	14	0.125	0.815 (0.381-1.745)	0.599	0.811 (0.379-1.738)	0.590
G/G-G/G	5	0.044	8	0.071	1.677 (0.531-5.292)	0.378	1.703 (0.538-5.393)	0.365
**c. 47C>T – *SOD2* (rs4880) and c.1823 C>T (p. Ser608Leu) – *NOS2* (rs2297518)**								
T/T-T/T	1	0.009	0	0.000	0.000 (0.000-+inf)	0.991	0.000 (0.000-+inf)	0.991
T/T-T/C	9	0.079	3	0.027	0.321 (0.085-1.219)	0.095	0.324 (0.085-1.234)	0.099
T/T-C/C	19	0.167	13	0.116	0.657 (0.307-1.403)	0.278	0.657 (0.307-1.405)	0.279
T/C-T/T	1	0.009	2	0.018	2.055 (0.184-22.985)	0.559	2.000 (0.177-22.536)	0.575
T/C-T/C	18	0.158	21	0.188	1.231 (0.616-2.458)	0.556	1.243 (0.621-2.489)	0.538
T/C-C/C	37	0.325	47	0.420	1.505 (0.875-2.589)	0.140	1.498 (0.869-2.581)	0.146
C/C-T/T	1	0.009	2	0.018	2.055 (0.184-22.985)	0.559	2.000 (0.177-22.536)	0.575
C/C-T/C	8	0.070	6	0.054	0.750 (0.252-2.236)	0.606	0.757 (0.253-2.259)	0.617
C/C-C/C	20	0.175	18	0.161	0.900 (0.448-1.809)	0.767	0.896 (0.445-1.801)	0.757
**c. 47C>T – *SOD2* (rs4880) and g.-1026 C>A – *NOS2* (rs2779249)**								
T/T-C/C	16	0.140	10	0.089	0.600 (0.260-1.387)	0.233	0.603 (0.261-1.395)	0.237
T/T-C/A	11	0.096	6	0.054	0.530 (0.189-1.486)	0.227	0.533 (0.190-1.497)	0.233
T/T-A/A	2	0.018	0	0.000	0.000 (0.000-+inf)	0.991	0.000 (0.000-+inf)	0.991
T/C-C/C	27	0.237	32	0.286	1.289 (0.711-2.338)	0.403	1.279 (0.703-2.327)	0.420
T/C-C/A	25	0.219	32	0.286	1.424 (0.778-2.605)	0.251	1.432 (0.782-2.622)	0.245
T/C-A/A	4	0.035	6	0.054	1.557 (0.527-5.672)	0.502	1.568 (0.430-5.720)	0.496
C/C-C/C	14	0.123	15	0.134	1.105 (0.506-2.410)	0.803	1.098 (0.503-2.398)	0.814
C/C-C/A	15	0.132	9	0.080	0.577 (0.241-1.378)	0.216	0.579 (0.242-1.384)	0.219
C/C-A/A	0	0.000	2	0.018	4149973.202 (0.000-+inf.)	0.991	4058473.181 (0.000-+inf.)	0.991
**c. 47C>T – *SOD2* (rs4880) and c.+396 T>G – *IL-8* (rs2227307)**								
T/T-T/T	7	0.061	5	0.045	0.714 (0.220-2.321)	0.576	0.724 (0.222-2.364)	0.593
T/T-T/G	15	0.132	9	0.080	0.577 (0.241-1.378)	0.216	0.582 (0.243-1.395)	0.225
T/T-G/G	7	0.061	2	0.018	0.278 (0.057-1.368)	0.115	0.269 (0.054-1.332)	0.108
T/C-T/T	14	0.123	14	0.125	1.020 (0.462-2.252)	0.960	1.004 (0.452-2.229)	0.992
T/C-T/G	29	0.254	38	0.339	1.505 (0.847-2.675)	0.163	1.512 (0.850-2.688)	0.159
T/C-G/G	13	0.114	18	0.161	1.488 (0.691-3.203)	0.310	1.492 (0.693-3.213)	0.307
C/C-T/T	6	0.053	9	0.080	1.573 (0.541-4.575)	0.406	1.585 (0.544-4.616)	0.398
C/C-T/G	17	0.149	12	0.107	0.685 (0.311-1.509)	0.347	0.673 (0.304-1.498)	0.328
C/C-GG	6	0.053	5	0.045	0.841 (0.249-2.839)	0.780	0.851 (0.251-2.878)	0.795
**c.+396 T>G – *IL-8* (rs2227307) and g.-1026 C>A – *NOS2* (rs2779249)**								
T/T-C/C	13	0.114	14	0.125	1.110 (0.496-2.481)	0.799	1.110 (0.497-2.482)	0.799
T/T-C/A	13	0.114	13	0.116	1.020 (0.451-2.310)	0.962	1.018 (0.449-2.305)	0.967
T/T-A/A	1	0.009	1	0.009	1.018 (0.063-16.478)	0.990	0.987 (0.061-16.070)	0.992
T/G-C/C	33	0.289	29	0.259	0.858 (0.478-1.540)	0.607	0.846 (0.469-1.525)	0.578
T/G-C/A	25	0.219	26	0.232	1.076 (0.577-2.009)	0.817	1.094 (0.582-2.054)	0.780
T/G-A/A	3	0.026	4	0.036	1.370 (0.300-6.267)	0.685	1.384 (0.302-6.339)	0.675
G/G-C/C	11	0.096	14	0.125	1.338 (0.579-3.088)	0.496	1.342 (0.581-3.100)	0.491
G/G-C/A	13	0.114	8	0.071	0.598 (0.238-1.503)	0.274	0.594 (0.236-1.496)	0.269
G/G-A/A	2	0.018	3	0.027	1.541 (0.253-9.404)	0.639	1.552 (0.254-9.479)	0.634
**c.+396 T>G – *IL-8* (rs2227307) and c.1823 C>T (p. Ser608Leu) – *NOS2* (rs2297518)**								
T/T-T/T	0	0.000	3	0.027	4188046.453 (0.000-+inf.)	0.989	4114068.704 (0.000-+inf.)	0.989
T/T-T/C	8	0.070	6	0.054	0.750 (0.252-2.236)	0.606	0.757 (0.253-2.259)	0.617
T/T-C/C	19	0.167	19	0.170	1.022 (0.509-2.051)	0.952	1.017 (0.506-2.043)	0.963
T/G-T/T	2	0.018	1	0.009	0.505 (0.045-5.644)	0.579	0.504 (0.045-5.646)	0.579
T/G-T/C	16	0.140	18	0.161	1.173 (0.565-2.435)	0.669	1.184 (0.569-2.464)	0.651
T/G-C/C	43	0.377	40	0.357	0.917 (0.534-1.576)	0.775	0.915 (0.532-1.572)	0.747
G/G-T/T	1	0.009	0	0.000	0.000 (0.000-+inf.)	0.991	0.000 (0.000-+inf.)	0.991
G/G-T/C	11	0.096	6	0.054	0.530 (0.189-1.486)	0.227	0.535 (0.190-1.505)	0.236
G/G-C/C	14	0.123	19	0.170	1.459 (0.692-3.077)	0.321	1.451 (0.688-3.063)	0.328
**c.+396 T>G – *IL-8* (rs2227307) and c.3331 G>A – *IL-6* (rs2069845)**								
T/T-G/G	4	0.035	11	0.098	2.995 (0.924-9.706)	0.067	2.980 (0.919-9.665)	0.069

T/T-G/A	12	0.105	12	0.107	1.020 (0.438-2.378)	0.963	1.011 (0.433-2.362)	0.979
T/T-A/A	11	0.096	5	0.045	0.438 (0.147-1.303)	0.138	0.441 (0.148-1.315)	0.142
T/G-G/G	14	0.123	13	0.116	0.938 (0.420-2.097)	0.876	0.934 (0.418-2.090)	0.869
T/G-G/A	31	0.272	35	0.313	1.217 (0.685-2.161)	0.503	1.229 (0.690-2.188)	0.483
T/G-A/A	16	0.140	11	0.098	0.667 (0.295-1.509)	0.331	0.661 (0.292-1.498)	0.321
G/G-G/G	8	0.070	5	0.045	0.619 (0.196-1.954)	0.414	0.622 (0.197-1.964)	0.418
G/G-G/A	13	0.114	12	0.107	0.932 (0.406-2.142)	0.869	0.923 (0.401-2.125)	0.851
G/G-A/A	5	0.044	8	0.071	1.677 (0.531-5.292)	0.378	1.703 (0.538-5.393)	0.365
**c.3331 G>A – *IL-6* (rs2069845) and g.-1026 C>A – *NOS2* (rs2779249)**								
G/G-C/C	12	0.105	18	0.161	1.628 (0.744-3.559)	0.222	1.623 (0.742-3.550)	0.225
G/G-C/A	13	0.114	11	0.098	0.846 (0.362-1.978)	0.700	0.846 (0.362-1.978)	0.700
G/G-A/A	1	0.009	0	0.000	0.000 (0.000-+inf.)	0.991	0.000 (0.000-+inf.)	0.991
G/A-C/C	30	0.263	27	0.241	0.889 (0.488-1.622)	0.702	0.873 (0.475-1.604)	0.662
G/A-C/A	24	0.211	27	0.241	1.191 (0.638-2.225)	0.583	1.213 (0.645-2.280)	0.549
G/A-A/A	2	0.018	5	0.045	2.617 (0.497-13.778)	0.256	2.646 (0.502-13.956)	0.251
A/A-C/C	15	0.132	12	0.107	0.792 (0.252-1.777)	0.572	0.800 (0.355-1.801)	0.590
A/A-C/A	14	0.123	9	0.080	0.624 (0.259-1.507)	0.295	0.619 (0.256-1.496)	0.286
A/A-A/A	3	0.026	3	0.027	1.018 (0.201-5.156)	0.982	1.019 (0.201-5.159)	0.982
**c.3331 G>A – *IL-6* (rs2069845) and c.1823 C>T (p. Ser608Leu) – *NOS2* (rs2297518)**								
G/G-T/T	1	0.009	1	0.009	1.018 (0.063-16.478)	0.990	0.987 (0.061-16.070)	0.992
G/G-T/C	9	0.079	7	0.063	0.778 (0.279-2.166)	0.631	0.785 (0.281-2.191)	0.644
G/G-C/C	16	0.140	21	0.188	1.413 (0.695-2.876)	0.340	1.405 (0.690-2.863)	0.348
G/A-T/T	1	0.009	1	0.009	1.018 (0.063-16.478)	0.990	0.987 (0.061-16.070)	0.992
G/A-T/C	17	0.149	15	0.134	0.882 (0.417-1.866)	0.743	0.894 (0.420-1.901)	0.771
G/A-C/C	38	0.333	43	0.384	1.246 (0.723-2.149)	0.428	1.239 (0.718-2.140)	0.441
A/A-T/T	1	0.009	2	0.018	2.055 (0.184-22.985)	0.559	2.056 (0.184-23.008)	0.559
A/A-T/C	9	0.079	8	0.071	0.897 (0.333-2.416)	0.830	0.899 (0.334-2.421)	0.834
A/A-C/C	22	0.193	14	0.125	0.597 (0.288-1.237)	0.165	0.599 (0.289-1.241)	0.168
