# Supplementary material for: Polymorphic variations and mRNA expression of the genes encoding interleukins as well as enzymes of oxidative and nitrative stresses as a potential risk of nephrolithiasis development
Source: PLoS One. 2023 Oct 25;18(10):e0293280. doi: 10.1371/journal.pone.0293280 (PMC10599546; doi:10.1371/journal.pone.0293280)
Supplement: S2 Table — (PDF) [file pone.0293280.s006.pdf]

**S4 Table.** Distribution of genotypes and alleles of the -597 A>G – *IL-6* (rs1800797), c.3331 G>A – *IL-6* (rs2069845), c.+396 T>G – *IL-8* (rs2227307), c.1823 C>T (p. Ser608Leu) – *NOS2* (rs2297518), g.-1026 C>A – *NOS2* (rs2779249) and ORs with 95% CIs in non-smokers and smokers.

| Genotypes/Alleles | NON-SMOKER (n = 127) | | | | SMOKER (n = 99) | | | |
|---|---|---|---|---|---|---|---|---|
| | Control (n = 68) | Urolithiasis (n = 59) | Crude OR (95% CI)* | *p* | Control (n = 46) | Urolithiasis (n = 53) | Crude OR (95% CI)* | *p* |
| | N (Freq.) | N (Freq.) | | | N (Freq.) | N (Freq.) | | |
| **-597 A>G – *IL-6* (rs1800797)** | | | | | | | | |
| A/A | 15 (0.221) | 15 (0.254) | 1.205 (0.531-2.734) | 0.656 | 8 (0.174) | 13 (0.245) | 1.544 (0.576-4.139) | 0.388 |
| A/G | 32 (0.471) | 30 (0.508) | 1.164 (0.579-2.339) | 0.670 | 26 (0.565) | 27 (0.509) | 0.799 (0.361-1.767) | 0.579 |
| G/G | 21 (0.309) | 14 (0.237) | 0.696 (0.316-1.535) | 0.369 | 12 (0.261) | 13 (0.245) | 0.921 (0.371-2.283) | 0.859 |
| | $\chi^2$ = 126.994; *p* = 0.409 | | | | $\chi^2$ = 98.992; *p* = 0.397 | | | |
| A | 62 (0.456) | 60 (0.508) | 1.230 (0.753-2.009) | 0.409 | 42 (0.457) | 53 (0.500) | 1.207 (0.674-2.163) | 0.527 |
| G | 74 (0.544) | 58 (0.492) | 0.813 (0.498-1.328) | 0.409 | 50 (0.543) | 53 (0.500) | 0.828 (0.462-1.484) | 0.527 |
| **c.3331 G>A – *IL-6* (rs2069845)** | | | | | | | | |
| G/G | 17 (0.250) | 15 (0.254) | 1.023 (0.458-2.283) | 0.956 | 9 (0.196) | 14 (0.264) | 1.476 (0.570-3.818) | 0.422 |
| G/A | 31 (0.456) | 32 (0.542) | 1.415 (0.702-2.849) | 0.332 | 25 (0.543) | 27 (0.509) | 0.872 (0.395-1.925) | 0.735 |
| A/A | 20 (0.294) | 12 (0.203) | 0.613 (0.270-1.393) | 0.242 | 12 (0.261) | 12 (0.226) | 0.829 (0.330-2.081) | 0.690 |
| | $\chi^2$ = 126.986; *p* = 0.409 | | | | $\chi^2$ = 98.994; *p* = 0.397 | | | |
| G | 65 (0.478) | 62 (0.525) | 1.208 (0.738-1.979) | 0.453 | 43 (0.467) | 55 (0.519) | 1.243 (0.698-2.213) | 0.459 |
| A | 71 (0.522) | 56 (0.475) | 0.828 (0.505-1.356) | 0.453 | 49 (0.533) | 51 (0.481) | 0.804 (0.452-1.432) | 0.459 |
| **c.+396 T>G – *IL-8* (rs2227307)** | | | | | | | | |
| T/T | 14 (0.206) | 15 (0.254) | 1.315 (0.573-3.016) | 0.518 | 13 (0.283) | 13 (0.245) | 0.825 (0.337-2.022) | 0.674 |
| T/G | 40 (0.588) | 30 (0.508) | 0.724 (0.359-1.462) | 0.368 | 21 (0.457) | 29 (0.547) | 1.438 (0.651-3.180) | 0.369 |
| G/G | 14 (0.206) | 14 (0.237) | 1.200 (0.518-2.779) | 0.670 | 12 (0.261) | 11 (0.208) | 0.742 (0.291-1.890) | 0.532 |
| | $\chi^2$ = 127.001; *p* = 0.409 | | | | $\chi^2$ = 99.000; *p* = 0.397 | | | |
| T | 68 (0.500) | 60 (0.508) | 1.038 (0.617-1.748) | 0.887 | 47 (0.511) | 55 (0.519) | 1.033 (0.589-1.812) | 0.910 |
| G | 68 (0.500) | 58 (0.492) | 0.963 (0.572-1.621) | 0.887 | 45 (0.489) | 51 (0.481) | 0.969 (0.552-1.698) | 0.910 |
| **c.1823 C>T (p. Ser608Leu) – *NOS2* (rs2297518)** | | | | | | | | |
| C/C | 46 (0.676) | 42 (0.712) | 1.182 (0.553-2.523) | 0.666 | 30 (0.652) | 36 (0.679) | 1.129 (0.489-2.609) | 0.776 |
| C/T | 20 (0.294) | 14 (0.237) | 0.747 (0.337-1.653) | 0.471 | 15 (0.326) | 16 (0.302) | 0.894 (0.382-2.093) | 0.796 |
| T/T | 2 (0.029) | 3 (0.051) | 1.768 (0.285-10.958) | 0.540 | 1 (0.022) | 1 (0.019) | 0.865 (0.053-14.236) | 0.919 |
| | $\chi^2$ = 127.001; *p* = 0.409 | | | | $\chi^2$ = 99.000; *p* = 0.397 | | | |

| | | | | | | | | |
|---|---|---|---|---|---|---|---|---|
| T | 24 (0.176) | 20 (0.169) | 0.955 (0.508-1.197) | 0.887 | 17 (0.185) | 18 (0.170) | 0.895 (0.418-1.915) | 0.775 |
| C | 112 (0.824) | 98 (0.831) | 1.047 (0.556-1.970) | 0.887 | 75 (0.815) | 88 (0.830) | 1.118 (0.522-2.392) | 0.775 |
| **g.-1026 C>A – *NOS2* (rs2779249)** | | | | | | | | |
| C/C | 30 (0.441) | 31 (0.525) | 1.402 (0.696-1.825) | 0.344 | 27 (0.587) | 26 (0.491) | 0.678 (0.306-1.503) | 0.338 |
| C/A | 34 (0.500) | 26 (0.441) | 0.788 (0.391-1.587) | 0.504 | 17 (0.370) | 21 (0.396) | 1.119 (0.496-2.525) | 0.786 |
| A/A | 4 (0.059) | 2 (0.034) | 0.561(0.099-3.181) | 0.514 | 2 (0.043) | 6 (0.113) | 2.809 (0.538-14.657) | 0.221 |
| | $\chi^2$ = 126.993; $p$ = 0.409 | | | | | $\chi^2$ = 98.933; $p$ = 0.398 | | |
| C | 94 (0.691) | 88 (0.746) | 1.383 (0.755-2.535) | 0.294 | 71 (0.772) | 73 (0.689) | 0.661 (0.350-1.247) | 0.201 |
| A | 42 (0.309) | 30 (0.254) | 0.723 (0.395-1.325) | 0.294 | 21 (0.228) | 33 (0.311) | 1.513 (0.802-2.856) | 0.201 |
