# Supplementary material for: Polymorphic variations and mRNA expression of the genes encoding interleukins as well as enzymes of oxidative and nitrative stresses as a potential risk of nephrolithiasis development
Source: PLoS One. 2023 Oct 25;18(10):e0293280. doi: 10.1371/journal.pone.0293280 (PMC10599546; doi:10.1371/journal.pone.0293280)
Supplement: S3 Table — (PDF) [file pone.0293280.s007.pdf]

Supplementary Table 3. Distribution of genotypes and alleles of the -597 A>G – *IL-6* (rs1800797), c.3331 G>A – *IL-6* (rs2069845), c.+396 T>G – *IL-8* (rs2227307), c. 47 C>T – *SOD2* (rs4880), c.1823 C>T (p. Ser608Leu) – *NOS2* (rs2297518) and ORs with 95% CIs in men and women with urolithiasis.

Genotypes/Alleles	WOMEN (n = 75) Control (n = 39) N (Freq.)	Urolithiasis (n = 36) N (Freq.)	Crude OR (95% CI)*	p	MEN (n = 151) Control (n = 75) N (Freq.)	Urolithiasis (n = 76) N (Freq.)	Crude OR (95% CI)*	p
**-597 A>G – *IL-6* (rs1800797)**								
A/A	7 (0.179)	9 (0.250)	1.524 (0.501-4.636)	0.458	16 (0.213)	19 (0.250)	1.229 (0.576-2.623)	0.594
A/G	17 (0.436)	20 (0.556)	1.618 (0.649-4.029)	0.302	41 (0.547)	37 (0.487)	0.787 (0.415-1.491)	0.462
G/G	15 (0.385)	7 (0.194)	0.386 (0.135-1.101)	0.075	18 (0.240)	20 (0.263)	1.131 (0.542-2.361)	0.743
	χ^2 = 74.978; p = 0.382				χ^2 = 151.000; p = 0.416			
A	31 (0.397)	38 (0.528)	1.704 (0.878-3.306)	0.115	73 (0.487)	75 (0.493)	1.028 (0.650-1.627)	0.950
G	47 (0.603)	34 (0.472)	0.587 (0.302-1.139)	0.115	77 (0.513)	77 (0.507)	0.972 (0.614-1.539)	0.905
**c.3331 G>A – *IL-6* (rs2069845)**								
G/G	8 (0.205)	9 (0.250)	1.292 (0.437-3.816)	0.643	18 (0.240)	20 (0.263)	1.131 (0.542-2.361)	0.743
G/A	17 (0.436)	21 (0.583)	1.812 (0.725-4.529)	0.204	39 (0.520)	38 (0.500)	0.923 (0.488-1.748)	0.806
A/A	14 (0.359)	6 (0.167)	0.357 (0.120-1.066)	0.065	18 (0.240)	18 (0.237)	0.983 (0.465-2.078)	0.964
	χ^2 = 74.960; p = 0.383				χ^2 = 151.000; p = 0.416			
G	33 (0.423)	39 (0.542)	1.635 (0.841-3.181)	0.147	75 (0.500)	78 (0.513)	1.055 (0.669-1.665)	0.817
A	45 (0.577)	33 (0.458)	0.612 (0.314-1.190)	0.147	75 (0.500)	74 (0.487)	0.948 (0.601-1.495)	0.817
**c.+396 T>G – *IL-8* (rs2227307)**								
T/T	9 (0.231)	8 (0.222)	0.952 (0.323-2.812)	0.930	18 (0.240)	20 (0.263)	1.131 (0.542-2.361)	0.743
T/G	21 (0.538)	20 (0.556)	1.071 (0.431-2.662)	0.882	40 (0.533)	39 (0.513)	0.922 (0.487-1.747)	0.804
G/G	9 (0.231)	8 (0.222)	0.952 (0.323-2.812)	0.930	17 (0.227)	17 (0.224)	0.983 (0.458-2.110)	0.965
	χ^2 = 75.000; p = 0.381				χ^2 = 151.000; p = 0.416			
T	39 (0.500)	36 (0.500)	1.000 (0.510-1.960)	1.000	76 (0.507)	79 (0.520)	1.056 (0.665-1.678)	0.816
G	39 (0.500)	36 (0.500)	1.000 (0.510-1.960)	1.000	74 (0.493)	73 (0.480)	0.947 (0.596-1.503)	0.816
**c. 47 C>T – *SOD2* (rs4880)**								
T/T	12 (0.308)	6 (0.167)	0.700 (0.234-2.091)	0.523	17 (0.227)	10 (0.132)	0.982 (0.471-2.049)	0.962
C/T	17 (0.436)	23 (0.639)	2.290 (0.904-5.797)	0.081	39 (0.520)	47 (0.618)	1.496 (0.783-2.859)	0.223
C/C	10 (0.256)	7 (0.194)	0.450 (0.148-1.365)	0.158	19 (0.253)	19 (0.250)	0.517 (0.219-1.218)	0.131
	χ^2 = 74.991; p = 0.382				χ^2 = 151.000; p = 0.416			

T	41 (0.526)	35 (0.486)	1.185 (0.609-2.306)	0.617	73 (0.487)	67 (0.441)	1.242 (0.759-2.031)	0.388
C	37 (0.474)	37 (0.514)	0.844 (0.434-1.642)	0.617	77 (0.513)	85 (0.559)	0.805 (0.492-1.317)	0.388
**c.1823 C>T (p. Ser608Leu) – *NOS2* (rs2297518)**								
C/C	22 (0.564)	24 (0.667)	1.545 (0.605-3.950)	3.636	54 (0.720)	54 (0.711)	0.955 (0.471-1.936)	0.897
T/C	16 (0.410)	12 (0.333)	0.719 (0.280-1.844)	0.492	19 (0.253)	18 (0.237)	0.915 (0.436-1.921)	0.814
T/T	1 (0.026)	0 (0.000)	0.000 (0.000-+inf.)	0.994	2 (0.027)	4 (0.053)	2.028 (0.360-11.419)	0.423
	χ^2 = 74.945; *p* = 0.383				χ^2 = 150.998; *p* = 0.416			
T	18 (0.231)	12 (0.167)	0.614 (0.251-1.502)	0.285	23 (0.153)	26 (0.171)	1.126 (0.627-2.022)	0.691
C	60 (0.769)	60 (0.833)	1.629 (0.666-3.984)	0.285	127 (0.847)	126 (0.829)	0.888 (0.495-1.594)	0.691
