# Supplementary material for: Polymorphic variations and mRNA expression of the genes encoding interleukins as well as enzymes of oxidative and nitrative stresses as a potential risk of nephrolithiasis development
Source: PLoS One. 2023 Oct 25;18(10):e0293280. doi: 10.1371/journal.pone.0293280 (PMC10599546; doi:10.1371/journal.pone.0293280)
Supplement: S4 Table — (PDF) [file pone.0293280.s008.pdf]

S2 Table. Distribution of haplotypes of the studied polymorphisms of the *IL-6* or *NOS2* genes and risk of urolithiasis.

Haplotypes	Control (n = 114)		Urolithiasis (n = 112)		Crude OR (95% CI)	*p*
	Number	Frequency	Number	Frequency		
**-597 A>G – *IL-6* (rs1800797) and c.3331 G>A – *IL-6* (rs2069845)**						
AG	104	0.456	111	0.495	1.171 (0.809-1.694)	0.401
GA	120	0.526	105	0.468	0.794 (0.548-1.148)	0.221
AA	4	0.017	6	0.026	1.541 (0.429-5.537)	0.504
GG	0	0.000	2	0.008	-	-
**c.1823 C>T (p. Ser608Leu) – *NOS2* (rs2297518) and g.-1026 C>A – *NOS2* (rs2779249)**						
TA	30	0.116	26	0.116	0.866 90.494-1.518)	0.616
CC	154	0.665	149	0.665	0.954 (0.644-1.413)	0.816
CA	33	0.165	37	0.165	1.169 (0.701-1.947)	0.548
TC	11	0.0.053	12	0.053	1.116 (0.482-2.585)	0.796
