# Supplementary material for: Polymorphic variations and mRNA expression of the genes encoding interleukins as well as enzymes of oxidative and nitrative stresses as a potential risk of nephrolithiasis development
Source: PLoS One. 2023 Oct 25;18(10):e0293280. doi: 10.1371/journal.pone.0293280 (PMC10599546; doi:10.1371/journal.pone.0293280)
Supplement: S5 Table — (PDF) [file pone.0293280.s009.pdf]

Supplementary Table 5. Distribution of genotypes and alleles of the -597 A>G – *IL-6* (rs1800797), c.3331 G>A – *IL-6* (rs2069845), c.+396 T>G – *IL-8* (rs2227307), c.1823 C>T (p. Ser608Leu) – *NOS2* (rs2297518), g.-1026 C>A – *NOS2* (rs2779249) and ORs with 95% CIs in subjects with normal body weight or subjects with overweight and obesity.

Genotypes/Alleles	BMI < 25 (n = 75)				BMI ≥ 25 (n = 151)			
	Control (n = 39)	Urolithiasis (n = 36)	Crude OR (95% CI)*	*p*	Control (n = 75)	Urolithiasis (n = 76)	Crude OR (95% CI)*	*p*
	N (Freq.)	N (Freq.)			N (Freq.)	N (Freq.)		
**-597 A>G – *IL-6* (rs1800797)**								
A/A	10 (0.256)	10 (0.278)	1.115 (0.401-3.105)	0.834	13 (0.173)	18 (0.237)	1.480 (0.666-3.288)	0.336
A/G	16 (0.410)	16 (0.444)	1.150 (0.460-2.875)	0.765	42 (0.560)	41 (0.539)	0.920 (0.485-1.748)	0.800
G/G	13 (0.333)	10 (0.278)	0.769 (0.287-2.065)	0.603	20 (0.267)	17 (0.224)	0.792 (0.377-1.667)	0.540
	χ^2 = 74.999; *p* = 0.381				χ^2 = 150.999; *p* = 0.416			
A	36 (0.462)	36 (0.500)	1.144 (0.628-2.086)	0.660	68 (0.453)	77 (0.507)	1.269 (0.786-2.050)	0.330
G	42 (0.538)	36 (0.500)	0.874 (0.479-1.593)	0.660	82 (0.547)	75 (0.493)	0.788 (0.488-1.273)	0.330
**c.3331 G>A – *IL-6* (rs2069845)**								
G/G	11 (0.282)	10 (0.278)	0.979 (0.357-2.686)	0.967	15 (0.200)	19 (0.250)	1.333 (0.619-2.874)	0.463
G/A	15 (0.385)	16 (0.444)	1.280 (0.510-3.125)	0.599	41 (0.547)	43 (0.566)	1.081 (0.569-2.054)	0.813
A/A	13 (0.333)	10 (0.278)	0.769 (0.287-2.065)	0.603	19 (0.253)	14 (0.184)	0.666 (0.305-1.451)	0.306
	χ^2 = 74.999; *p* = 0.381				χ^2 = 151.001; *p* = 0.416			
G	37 (0.474)	36 (0.500)	1.092 (0.604-1.974)	0.772	71 (0.473)	81 (0.533)	1.310 (0.808-2.124)	0.273
A	41 (0.526)	36 (0.500)	0.916 (0.507-1.657)	0.772	79 (0.527)	71 (0.467)	0.763 (0.471-1.238)	0.273
**c.+396 T>G – *IL-8* (rs2227307)**								
T/T	8 (0.205)	9 (0.250)	1.292 (0.437-3.816)	0.643	19 (0.253)	19 (0.250)	0.982 (0.417-2.049)	0.962
T/G	19 (0.487)	18 (0.500)	1.053 (0.425-2.605)	0.912	42 (0.560)	41 (0.539)	0.920 (0.485-1.748)	0.800
G/G	12 (0.308)	9 (0.250)	0.750 (0.272-2.071)	0.579	14 (0.187)	16 (0.211)	1.162 (0.522-2.588)	0.713
	χ^2 = 75.000; *p* = 0.381				χ^2 = 151.000; *p* = 0.416			
T	35 (0.449)	36 (0.500)	1.227 (0.646-2.329)	0.533	80 (0.533)	79 (0.520)	0.941 (0.584-1.516)	0.803
G	43 (0.551)	36 (0.500)	0.815 (0.429-1.548)	0.533	70 (0.467)	73 (0.480)	1.063 (0.659-1.712)	0.803
**c.1823 C>T (p. Ser608Leu) – *NOS2* (rs2297518)**								

C/C	27 (0.692)	28 (0.778)	1.556 (0.550-4.397)	0.405	49 (0.653)	50 (0.658)	1.020 (0.521-1.997)	0.953
C/T	11 (0.282)	6 (0.167)	0.509 (0.166-1.561)	0.237	24 (0.320)	24 (0.316)	0.981 (0.494-1.946)	0.956
T/T	1 (0.026)	2 (0.056)	2.235 (0.194-25.765)	0.519	2 (0.027)	2 (0.026)	0.986 (0.135-7.191)	0.989
	χ^2 = 75.013; p = 0.381				χ^2 = 151.000; p = 0.416			
T	13 (0.167)	10 (0.139)	0.826 (0.355-1.923)	0.657	28 (0.187)	28 (0.184)	0.983 (0.542-1.784)	0.955
C	65 (0.833)	62 (0.861)	1.211 (0.520-2.819)	0.657	122 (0.813)	124 (0.816)	1.017 (0.560-1.847)	0.955
**g.-1026 C>A – *NOS2* (rs2779249)**								
C/C	21 (0.538)	17 (0.472)	0.767 (0.309-1.901)	0.567	36 (0.480)	40 (0.526)	1.204 (0.636-2.280)	0.569
C/A	15 (0.385)	15 (0.417)	1.143 (0.453-2.881)	0.777	36 (0.480)	32 (0.421)	0.788 (0.414-1.498)	0.467
A/A	3 (0.077)	4 (0.111)	1.500 (0.312-7.216)	0.613	3 (0.040)	4 (0.053)	1.333 (0.288-6.171)	0.713
	χ^2 = 75.000; p = 0.381				χ^2 = 151.000; p = 0.416			
C	57 (0.731)	49 (0.681)	0.791 (0.395-1.585)	0.508	108 (0.720)	112 (0.737)	1.104 (0.639-1.907)	0.723
A	21 (0.269)	23 (0.319)	1.264 (0.631-2.534)	0.508	42 (0.280)	40 (0.263)	0.906 (0.524-1.565)	0.723
